# The Signature Microbiota Drive Rumen Function Shifts in Goat Kids Introduced to Solid Diet Regimes

**DOI:** 10.3390/microorganisms7110516

**Published:** 2019-10-31

**Authors:** Xiaokang Lv, Jianmin Chai, Qiyu Diao, Wenqin Huang, Yimin Zhuang, Naifeng Zhang

**Affiliations:** 1 Feed Research Institute, Chinese Academy of Agricultural Sciences, Key Laboratory of Feed Biotechnology of the Ministry of Agriculture, Beijing 100081, China; 13121191399@163.com (X.L.); jchai@uark.edu (J.C.); diaoqiyu@caas.cn (Q.D.); m13121271017@163.com (W.H.);; 2 Department of Animal Science, Division of Agriculture, University of Arkansas, Fayetteville, AR 72701, USA

**Keywords:** goats, rumen microbiota, solid diet, rumen development, neutral detergent fibers, volatile fatty acids

## Abstract

The feeding regime of early, supplementary solid diet improved rumen development and production in goat kids. However, the signature microbiota responsible for linking dietary regimes to rumen function shifts are still unclear. This work analyzed the rumen microbiome and functions affected by an early solid diet regime using a combination of machine learning algorithms. Volatile fatty acids (i.e., acetate, propionate and butyrate) fermented by microbes were found to increase significantly in the supplementary solid diet groups. Predominant genera were found to alter significantly from unclassified *Sphingobacteriaceae* (non-supplementary group) to *Prevotella* (supplementary solid diet groups). Random Forest classification model revealed signature microbiota for solid diet that positively correlated with macronutrient intake, and linearly increased with volatile fatty acid production. Bacteria associated with carbohydrate and protein metabolism were also identified. Utilization of a Fish Taco analysis portrayed a set of intersecting core species contributed to rumen function shifts by the solid diet regime. The core community structures consisted of the specific, signature microbiota and the manipulation of their symbiotic partners are manipulated by extra nutrients from concentrate and/or forage, and then produce more volatile fatty acids to promote rumen development and functions eventually host development. Our study provides mechanisms of the microbiome governed by a solid diet regime early in life, and highlights the signature microbiota involved in animal health and production.

## 1. Introduction

The development of advanced genomic research techniques such as the introduction of next- generation sequencing and its downstream analysis has allowed a deeper investigation of the gut microbiome. Early life diet is an important driver in shaping the long-term and adult gut microbiome profiles due to the huge alteration of diet components and macronutrient levels, especially the introduction of a solid diet, compared to breast milk and gut development [1]. The goat rumen, with rapid physiological changes, such as non-rumination, transition and rumination, could be proposed as an appropriate animal model for studying the development of gut microbial ecosystems by early diet manipulation providing a means for prevention of metabolic diseases [2,3]. Young ruminants receiving only milk or fluid diets (milk replacer) have limited metabolic activity in the rumen epithelium and minimal absorption of volatile fatty acids (VFAs) [4]. Early supplementary feeding of a solid diet has already been widely used in lamb production to improve rumen and body development because of its stimulation of microbial proliferation and VFA production that initiates epithelial development [5]. A solid concentrate (starter) diet containing a high concentration of carbohydrates has been widely used to rear pre-weaned ruminants [4,6,7]. Compared with breast milk-fed lambs, the community structure and composition of the rumen microbiota in starter-fed lambs tends to mature more easily and quickly [8]. Lin and their colleagues [3] analyzed rumen microbiota in starter fed lambs vs breast-milk fed lambs. They found that acetate and butyrate increased in the rumen of starter-fed lambs, as well as increases of 5 genera including *Mitsuokella*, *Sharpea*, *Megasphaera*, *Dialiste*, and unclassified *Bifidobacteriaceae*. Supplementation of extra alfalfa on the basis of concentrate diets has also been noted to improve rumen development to the next level. Previous studies reported that increases of growth performance and changes of ruminal microbiota during the pre- and post-weaning periods were found in lambs fed starter plus alfalfa compared with lambs fed fluid-diet and starter [9,10]. In addition, studies have summarized the significant changes of microbiota in solid feeding regimes and evenly calculated the correlation between macronutrient intake and rumen bacterial abundances. Wang et al. [11] found a correlation between bacterial genera in lambs rumen tissue and functional variables of rumen samples sequenced on d42. Yang et al. [10] sequenced rumen samples from *Hu* lambs fed milk replacer from d5 to d38 and supplied with solid diet (starter and alfalfa). They also observed the effects of a solid diet on microbial composition, and a set of taxon correlated with crude protein (CP), neutral detergent fiber (NDF) and body weight.

Until now, these studies have remarkably shown extended effects of a solid diet on the development of rumen functions and microbial communities in lambs, however they mainly focus on the microbiota at weaning day (around d40) or at the genus microbial level. This leaves many important questions remaining unknown. One such uncertainty is considering whether goat kids have similar patterns when fed a solid diet, due to lambs and goats belonging to a different genus. Additionally, what are the signature microbiota for supplementary regimes? How does the regime supplemented starter with alfalfa affect manipulation of the rumen microbiota? How do the signature microbiota associated with other members in a solid diet regime maintain equilibrium and improve function? To more rigorously analyze these questions, a study that feeds goats with a solid supplement diet to investigate the microbiome and it’s association with experimental factors and rumen functions utilizing more in-depth machine learning algorithms is necessary. Random Forest, an ensemble learning method for classification and regression, can be used to rank the importance of predictive variables in a regression or classification problem naturally [12]. Another machine learning algorithm, Fish Taco is a computational framework for comprehensively computing taxon-level contribution to detected functional shifts and the identification of key taxa, was introduced by Manor et al. [13]. Network analyses that identify the microbial interaction allows us to characterize how the “core” microbiota impacts the overall composition and functions of the microbiome necessary for answer those important questions. [14].

Deep analysis of microbial data with a combination of the above three algorithms gives more in-depth insights of the microbiome. Examining the correlation between the microbiome and phonotype, such as macronutrient intake and rumen fermentation parameters, aids in the investigation of microbiome alterations and the subsequent rumen fermentation environment influenced by a solid diet regimen early in life. Therefore, the objectives of this work were to assess the rumen fermentation, microbiome community and function shifts influenced by the supplementation of a solid diet, fed until to d 60 (the rumination phase).

## 2. Materials and Methods

### 2.1. Goat Kids, Treatments and Management

The experimental procedures of this project were approved by the Chinese Academy of Agricultural Sciences Animal Ethics Committee, and humane animal care and husbandry procedures were followed throughout this study (Protocol Number: AEC-CAAS-FRI-CAAS20180305; Approval date: 9 March 2018). This animal trial was conducted using Haimen goat kids at a commercial farm in the Jiangsu province, China.

A total of 72 Haimen goat kids (20 days old and average body weight 4.54 ± 0.51 kg) were separated from their dams, and randomly allotted to three groups based on their following diets: milk replacer only (MRO), milk replacer + concentrate (MRC), milk replacer + concentrate + alfalfa pellets (MCA). Each treatment had six replicates and four kids per pen were as a replicate.

Goat kids remained with their mother and received breast milk from 0 to 20 days. During 20 to 60 days of age, they were separated with their dams and the above 3 diet treatments were provided to their corresponding groups. During animal trial, all goat kids had *ad libitum* access to water, MRC and MCA kids could freely access concentrate, and MCA goats were supplied the additional alfalfa pellets. Nutritional levels of milk replacer, concentrate and alfalfa pellets are shown in Table S1.

### 2.2. Sample Collection and Chemical Analysis

Daily feed intakes were recorded within each feeding treatment. CP, non-fiber carbohydrate (NFC), and NDF of each regimen sample was analyzed according to the Association of Official Analytical Chemists [15]. Subsequently average daily intake of CP, NFC and NDF were calculated. Table S1 (dietary composition) and Table S2 (growth performance) were published in a Chinese journal paper [16], with the remaining data such as rumen fermentation parameters and microbiome analysis detailed in this manuscript.

Six goat kids (healthy and BW close to the average BW of the corresponding groups) were selected from each group and euthanized for rumen sample collections. At 60 days of age, the goat kids were taken to an on-farm slaughterhouse, anesthetized using sodium pentobarbitone, and euthanized by exsanguination at the jugular vein. Next, rumen organs were removed and the ruminal content pH was measured immediately using a pH electrode (PB-10; Sartorius, Goettingen, Germany). Approximately 10 mL of rumen content was sampled from the mixed rumen digesta and stored at −80 °C for sequencing. Rumen fluid approximate the 10 mL level was filtered through four layers of gauze and placed in a 15 mL centrifuge tube and immediately frozen at −20 °C for the analysis of rumen fermentation. Determination of rumen fluid ammonia nitrogen (NH_3_-N) concentration by a phenol-sodium hypochlorite colorimetric method was performed after the liquid was thawed at 4 °C. Microbial proteins were analyzed according to the method described by Makkar et al. [17]. VFA concentration in the rumen fluid was quantified by gas chromatography (GC) [18] using methyl valerate as the internal standard in an Agilent 6890 series GC equipped with a capillary column (HP-FFAP19095F-123, 30 m, 0.53 mm diameter and 1 mm thickness, Agilent Technologies, Santa Clara, CA, USA).

### 2.3. DNA Extraction and 16S rRNA Gene Sequencing

Rumen fluid samples were thawed on ice and microbial DNA was extracted using a commercial DNA Kit (Omega Bio-tek, Norcross, GA, U.S.) according to the manufacturer’s instructions. Total DNA quality was analyzed using a NanoDrop 2000 UV spectrophotometer (ThermoFisher, Waltham, MA, USA) and 1% agarose gel electrophoresis. The V3–V4 region of the bacterial 16S ribosomal RNA genes were amplified by PCR (95 °C for 3 min, followed by 30 cycles at 98 °C for 20 s, 58 °C for 15 s, and 72 °C for 20 s and a final extension at 72 °C for 5 min) using indexes and adaptor-linked universal primers (431 F: ACTCCTACGGGRSGCAGCAG; 806R: GGACTACVVGGGTA TCTAATC). PCR reactions were performed in 30 μL mixtures containing 15 μL of 2 × KAPA Library Amplification Ready Mix, 1 μL of each primer (10 μM), and 50 ng of template DNA and ddH_2_O. All PCR products were normalized and quantified by a Qubit 2.0 Fluorometer (Thermo Fisher Scientific, Waltham, MA, USA). Amplicon libraries were mixed using all qualified products and sequenced with an Illumina HiSeq PE250 platform at Realbio Technology Genomics Institute (Shanghai, China).

### 2.4. Sequencing Data Processing

Raw sequences were filtered through a quality control pipeline using the Quantitative Insight into Microbial Ecology (QIIME v 1.9.1) tool kit [19]. The chimeras and singletons were detected and removed by Usearch software (v7.0.1090), and high-quality sequences were clustered into operational taxonomic units (OTUs) at the 97% similarity level. Samples were normalized to 24136 sequencing reads. The representative sequences were classified based on the Ribosomal Database Project (RDP) database [20] at the default confidence threshold of 0.8, trained on the SILVA reference database (release 111) [21]. The alpha diversities (Shannon Index and Observed species), and beta diversities (Unweighted and Weighted Unifrac distance) were additionally calculated. The ANalysis Of SIMilarity (ANOSIM) test was used to examine the statistically significant differences in beta diversity. The datasets in this study are available in the NCBI BioProject database with the BioProject ID PRJNA544381 (https://www.ncbi.nlm.nih.gov/sra/PRJNA544381).

### 2.5. Data Analysis

Rumen fermentation parameters were shown using bar charts made in R (v3.6.0) by the ‘ggplot2′ package. Anova tests were used for significance calculations after the detection of homogeneity of variance. Following the check of global test significance, a post-hoc analysis (Tukey’s HSD test) was performed to determine which groups of independent variables differ from the other groups.

Alpha diversity of the rumen microbial data among the three treatments was tested using the Kruskal–Wallis test and a post-hoc Dunn Kruskal-Wallis multiple comparison with a Bonferroni adjustment to evaluate differences between the two groupswith boxplots being made in R (‘ggpubr’ packages). Beta diversity was visualized with a PCoA plot.

The Random Forest classification model (AUCRF) based on optimization of the area under the receiver operating characteristic curve (AUC) was performed to identify the top microbiome signatures to differentiate the 3 supplementary feeding regimes. Variables importance plot was then generated based on the importance scores (Mean Decrease in Accuracy, MDA) of optimal features and their boxplots of selected features were drawn in R (v3.6.0).

Random Forest’s regression model was used to select the rumen microbiota that were important for the average daily intake of major nutrients (i.e., CP, NDF and NFC) and the rumen fermentation parameters. The top 50 selected features were then analyzed by a Pearson correlation with those macro indicators, respectively.

Functional Shifts’ Taxonomic Contributors (Fish Taco v1.1.1) software was used to identify the rumen bacteria driving the functional shifts between the supplementary feeding regimes in this study. A taxonomic abundance table at the OTUs’ level and functional abundance profiles at level 3 from the PICRUSt analysis were used. In pairwise comparisons, we labeled MRO groups as the control and MRC or MCA groups as the case, and tested the MRC treatment as the control with the MCA treatment as a case. Each functional shift was grouped into a case-associated with driving or an attenuating case-enrichment, and a control-associated driving or an attenuating case-enrichment. The output result visualization was performed in the Fish Taco Plot package in R (v 3.6.0).

To assess microbial interaction within treatments, network analysis was performed by calculating all possible Pearson rank correlation coefficients (ρ) between microbial pairs. To minimize the occurrence of spurious associations, we considered a valid co-occurrence between two different taxa, if a correlation co-efficiency was over 0.6 or less than 0.6 and was statistically significant. The subnetworks in regimes were produced based on the betweenness cluster calculated by the Girvan-Newman algorithm [22]. Further information regarding data processing and analysis is provided in the supplementary material. A reproducible version of this analysis and computer code is available at https://github.com/chaichai9521/goat-rumen-mcirobiome-analysis.git.

## 3. Results

### 3.1. Rumen Fermentation Parameters

**Figure 1:**
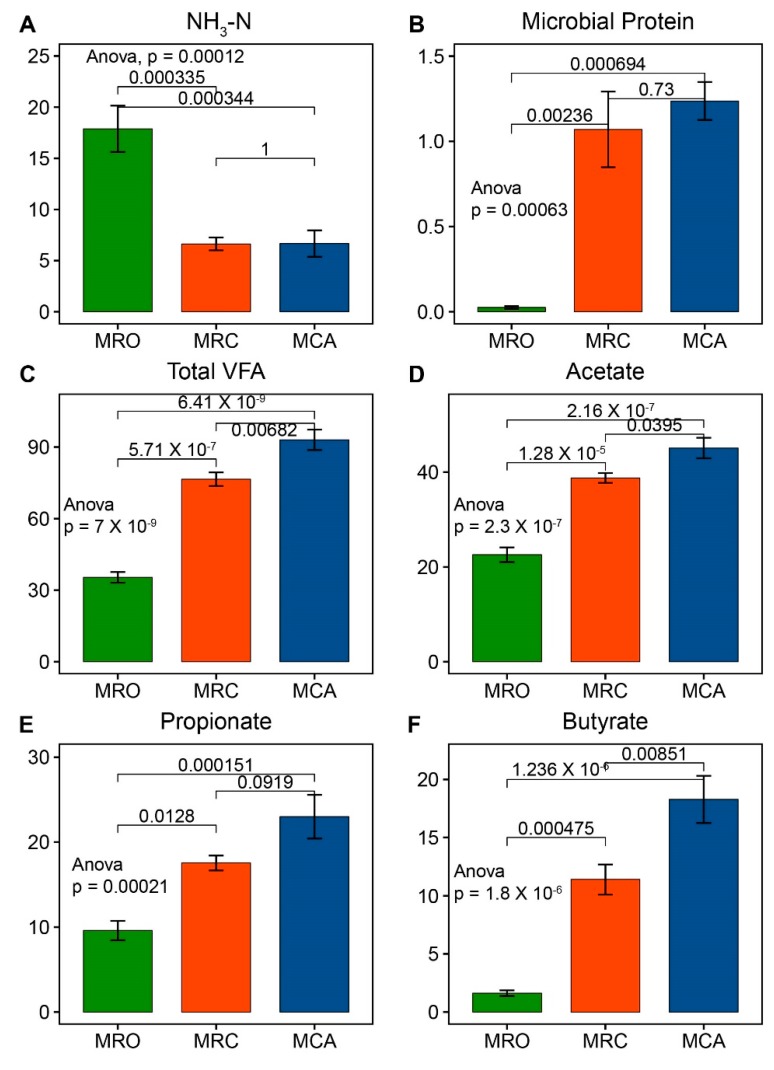
Effects of early supplementary solid diet on rumen fermentation parameters in goat kids. The significant differences of rumen NH_3_-N (**A**), microbial proteins (**B**), total VFA (**C**), acetate (**D**), propionate (**E**) and butyrate (**F**) were found. An Anova test was used for significance calculation after detection of homogeneity of variance. After the global test was significant, a post-hoc analysis (Tukey’s HSD test) was performed to determine which group of the independent variable differ from each other group. High dietary nitrogen conversion ratio was found in MRC and MCO (*p* < 0.05). The total VFA, acetate propionate and butyrate had the highest values in MCA (*p* < 0.05), and were significantly higher in MRC than in MRO (*p* < 0.05). MRO = milk replacer only, MRC = milk replacer + concentrate, MCA = milk replacer + concentrate + alfalfa. VFA: Volatile fatty acids. Statistical significance was accepted at *p* < 0.05.

Rumen fermentation parameters affected by the different dietary regimes were observed in this study (Figure 1 and Table S3). The MRO group had a greater concentration of NH_3_-N (*p* < 0.05) compared with the MRC and MCA groups, while the opposite patterns of ruminal microbial proteins were found. Higher concentrations of total VFA, acetate, propionate, butyrate and valerate in supplementary solid diet regimes (MRC, MCA) compared to MRO were observed (*p* < 0.05), and with the exception of propionate and valerate, it was found that acetate, butyrate and total VFA were higher in MCA than MRC (*p* < 0.05).

Since key factors in diets influenced the goat rumen environment and development were intake of nutrients including CP, NFC and NDF, a correlation between nutrient intake and rumen fermentation parameters was performed (Table S4). Regression analysis confirmed that pH and NH_3_-N were negatively associated with average daily intake of CP, NFC and NDF, while rumen MCP and VFA (i.e., acetate, propionate, butyrate, and Total VFA) concentrations had the strongest positive association with nutrient intake.

### 3.2. The Diversity and Core Bacteria in Rumen Microbiome

**Figure 2:**
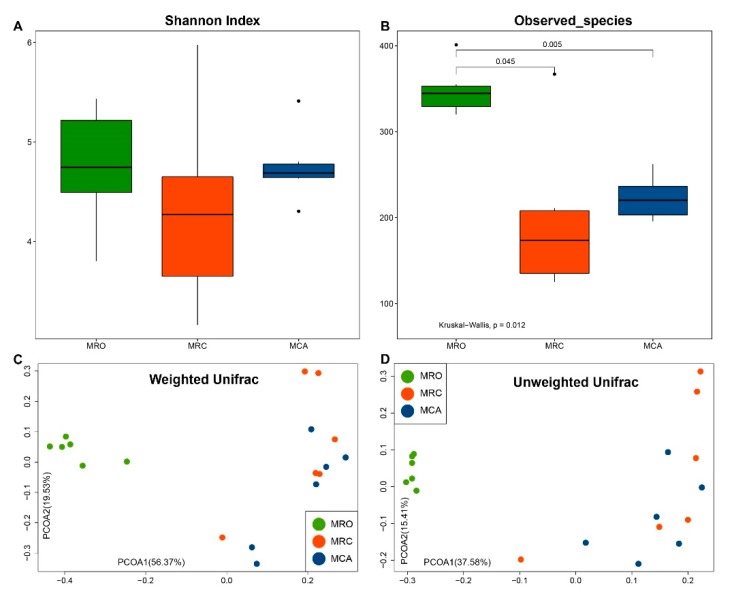
The early supplementary solid diet impacted on Alpha and Beta diversity of rumen microbiome in goat kids. (**A**,**B**) The Shannon Index and Observed species. Alpha diversity of the rumen microbial data were tested using the Kruskal-Wallis test and a post-hoc Dunn Kruskal-Wallis multiple comparison, and the Bonferroni method was used for *p* value correction. Principal coordinate analysis (PCoA) of the community membership based on the weighted (**C**) and unweighted (**D**) UniFrac distance are depicted with the green cycles as the MROgroup, the red cycles as the MRC group and the blue cycles as the MCA group. Although diversity (Shannon index) was not different (*p* = 0.372), significance of microbial richness was observed among MRO, MRC and MCA groups of rumen samples (*p* = 0.012). Significances in community structure among the 3 groups were detected (Weighted Unifrac ANOSIM, *R* = 0.68, *p* < 0.05; UnWeighted Unifrac ANOSIM, *R* = 0.69, *p* = 0.001). The MRO formed a distinct cluster on the left side, while the MRC and MCA were closely clustered on the right side of the PCoA plot. MRO = milk replacer; MRC = milk replacer + concentrate; MCA = milk replacer + concentrate + alfalfa; ANOSIM: Analysis of similarity.

After quality control, filtering and OTU clustering steps, 641,197 high quality sequencing reads across all samples, with an average of 35,622 sequencing reads for each sample, were generated. First, we analyzed the rumen content microbiome at the community level. Although diversity (Shannon Index) was not different (*p* = 0.372), significance of microbial richness was observed among the MRO, MRC and MCA rumen samples (*p* = 0.012) (Figure 2A,B). The MRO rumen microbiota had a significantly higher observed species than both MRC and MCA samples (*p* = 0.045, *p* = 0.005), and there was no difference between MRC and MCA (*p* = 0.180). The observed species of rumen microbiome was negatively correlated with nutrient average daily intake including CP (*r* = −0.65, *p* = 0.003), NFC (*r* = −0.73, *p* = 0.001) and NDF (*r* = −0.74, *p* = 0.0003) (Table S5). Negative associations between the microbial richness, MCP and VFA including acetate, propionate, butyrate, valerate and total VFA were also observed. Regarding beta diversity measurements, significant clusters in community structure among the three feeding regimes were detected (Weighted Unifrac ANOSIM, *R* = 0.68, *p* < 0.05; UnWeighted Unifrac ANOSIM, *R* = 0.69, *p* = 0.001). The MRO formed a distinct cluster (green dots) on the left side, while the MRC and MCA were closely clustered (red and blue dots) on the right side of the PCoA plot (Figure 2C,D).

We next examined the rumen core microbiome among the three treatments. At the genus level, a total of 152 genera were observed, and *Prevotella* followed by unclassified *Prevotellaceae*, unclassified *Sphingobacteriaceae* and unclassified *Bacteroidetes* accounted for 63.29% of the total sequences, indicating these microbials as the predominant genera with abundance over 5% across all samples (Figure S1). The top genera in the MRO group was unclassified *Sphingobacteriaceae* (30.32%), unclassified *Prevotellaceae* (16.92%), unclassified *Bacteroidetes* (11.77%) and *Prevotella* (8.91%). In the MRC group, *Prevotella* (56.02%) were the predominant bacteria, followed by *Roseburia* (4.49%), unclassified *Prevotellaceae* (4.29%), *Selenomonas* (3.82%) and unclassified *Lachnospiraceae* (3.73%). However, in the MCA group, the abundance of the predominant genus *Prevotella* (44.02%) decreased compared to the MRC group, and the other dominant genera were unclassified *Prevotellaceae* (11.63%), *Fibrobacter* (7.01%), *Treponema* (5.35%), *Succinivibrio* (4.74%) and unclassified *Lachnospiraceae* (4.50%).

**Figure 3:**
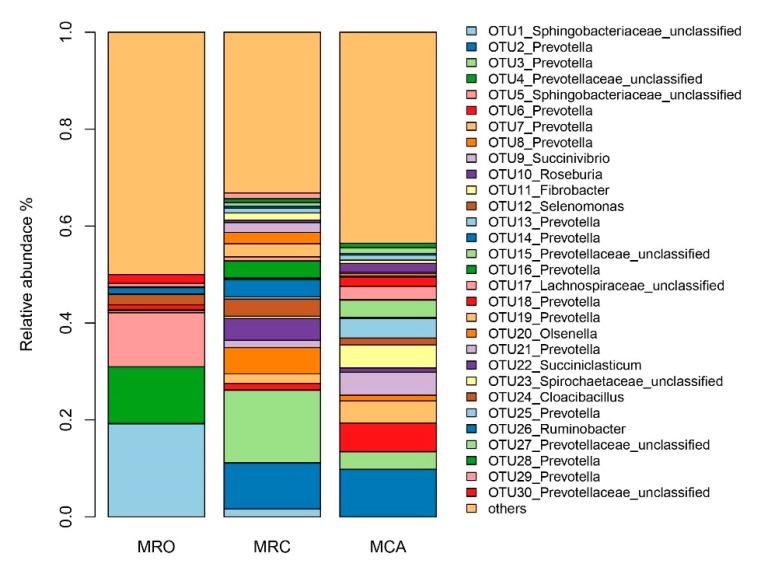
The top 30 OTUs in the three supplementary regimes. Each bar shows the average relative abundance of MRO, MRC and MCA groups. Each color represents the relative abundance of a bacterial taxon on the stacked bar chart. MRO = milk replacer, MRC = milk replacer + concentrate, MCA = milk replacer + concentrate + alfalfa.

At the OTU level, there were 281 OTUs that were significantly different between the three groups (Table S6), and 16 taxa in the top 30 were found significant. The top 30 most abundant bacterial taxa accounting for 57.77% of all reads are displayed on the stacked bar charts (Figure 3). Among the top 30 OTUs, 14 belong to the genus *Prevotella*, and 4 were in the genus unclassified *Prevotellaceae*. The OTUs belonging to unclassified *Sphingobacteriaceae* (OTU1 and OTU5), unclassified *Prevotellaceae* (OTU4 and OTU30) and *Cloacibacillus* (OTU24) were greater in the MRO group. The OTUs affiliated with *Prevotella* (OTU2, OTU6, OTU13,) in the top 30 had a higher abundance in the MRC and MCA groups. The MRC group was found abundant with *Roseburia* (OTU10), *Olsenella* (OTU20) and *Prevotella* (OTU21). The bacteria belonging to the genera *Prevotella* (OTU6 and OTU13) *Succinivibrio* (OTU9), unclassified *Prevotellaceae* (OTU15), *Succiniclasticum* (OTU22) had the highest relative abundances in the MCA group.

### 3.3. The Signature Microbiota Differentiating MRO, MRC and MCA Supplementary Regimes

**Figure 4:**
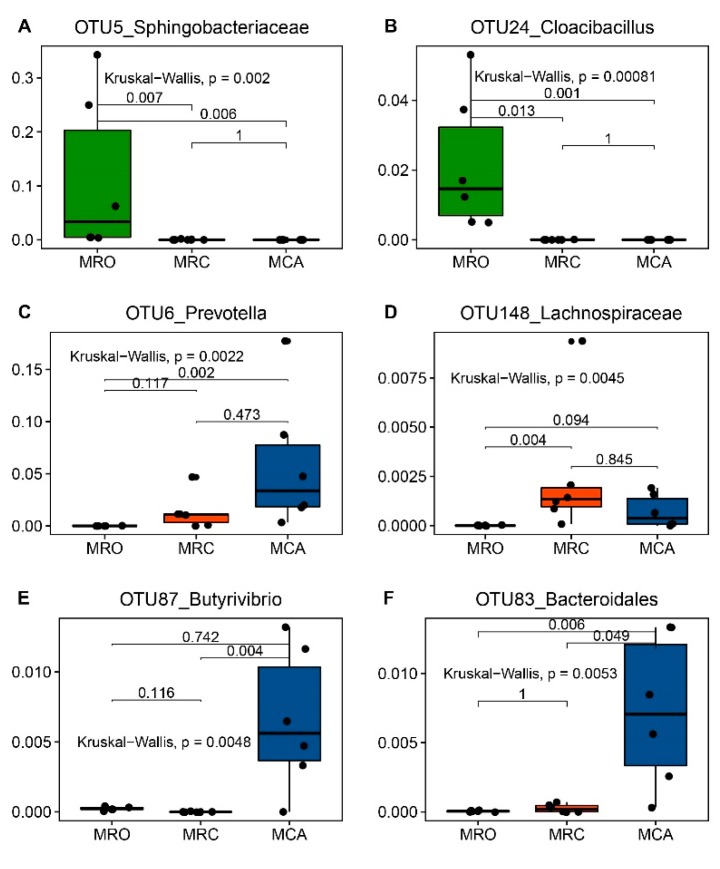
The highlight signature microbiota identified by AUCRF for differentiating MRO, MRC and MCA. The OTU5 (unclassified *Sphingobacteriaceae*), OTU24 (*Cloacibacillus*) had high abundances in MRO group (**A**,**B**). OTU148 (unclassified *Lachnospiraceae*) was higher in MRC and MCA (**D**). Other OTUs including OTU6 (*Prevotella*), OTU87 (*Butyrivibrio*) and OTU83 (unclassified *Bacteroidales*) were significantly enriched in the MCA (**C**,**E**,**F**). All the OTUs abundances were tested using the Kruskal–Wallis test and a post-hoc Dunn Kruskal-Wallis multiple comparison with the Bonferroni method for *p* value correction being used. The black dots within each bar were values from individual animals, and the black lines within each bar represented the medians. MRO = milk replacer; MRC = milk replacer + concentrate; MCA = milk replacer + concentrate + alfalfa; AUCRF: Random Forest based on optimizing the area-under-the receiver operator characteristic curve (AUC).

To identify the rumen important microbiome that differentiate MRO, MRC and MCA groups, we performed an updated Random Forest classification model to differentiate these 3 supplementary regimes. The regimes-associated bacterial features were listed based on their MDA and the representatively selected microbiota were presented in Figure 4. All three groups were analyzed together, and optimal features with an AUC of 1.00 (specificity 1.00, sensitivity 1.00) were selected from the AUCRF model (Table S7 and Figure S2). High AUC (0.931) was still observed at the 50th feature, suggesting those signatures could accurately predict whether goats were fed concentrate plus alfalfa or concentrate only. Among the top 50 features, only three core species, such as OTU5 (unclassified *Sphingobacteriaceae*), OTU24 (*Cloacibacillus*) and OTU6 (*Prevotella*), were identified as regime-associated bacteria (Figure 4).

Forty of the top 50 bacteria were more abundant in the MRO group. OTU5 associated with the MRO group was the predominant genus, with greaterrelative abundance and prevalence (11.13%; 6/6) compared to the MRC (0.03%, 2/6) and MCA (0.04%, 2/6). OTU24 concerning qualitative signatures had greater abundance, 2.16%, in the MRO. Other species associated with *Prevotella* that were enriched in the solid diet groups were found more abundant in the MRO group, including OTU119, OTU42 and OTU60. Considering the MCA group’s microbiome, OTU6 and OTU104 affiliated with the predominant *Prevotella* increased. We observed the relative abundances of OTU6 were 0.01%, 1.35% and 5.89% in the MRO, MRC and MCA groups (prevalence 2/6, 6/6 and 6/6). OTU87 (*Butyrivibrio*) and OTU83 (unclassified *Bacteroidales*) were significantly enriched in the MCA group, and extremely low abundance in the MRO and/or MRC groups was found. Similar patterns were found in the other MCA group predictors such as OTU93 and OTU74 (*Treponema*), OTU539 (unclassified *Clostridiales*), OTU396 (unclassified *Proteobacteria*), OTU221 (*Pseudobutyrivibrio*) and OTU110 (unclassified *Prevotellaceae*) (Figure S3).

We then performed pairwise AUCRF comparisons to validate these predictors. The results confirmed that most of the classified biomarkers could also be listed (Figures S4–S6). Moreover, the MRC group was enriched with OTU148 (unclassified *Lachnospiraceae*) and OTU114 (*Megasphaera*) compared to the MRO group, whereas more abundance of OTU643 (*Neisseria*), OTU177 (*Campylobacter*) and OTU314 (*Blautia*) is characterized compared with the MCA group.

### 3.4. Phenotypes and Rumen Microbiota

To characterize the relationship between the rumen microbiota and major nutrients in the diet to better understand how supplementary feeding regimes influenced microbial communities, the following was conducted. First, we performed a Random Forest regression model by using CP, NFC and NDF intake as outcomes and all taxa as independent variables. Then, the Pearson correlations were calculated between the selected top 50 bacterial abundances and dietary CP, NFC and NDF intakes, respectively (Table 1). Additionally, impacts of the alteration of the rumen microbiota on rumen VFA were also estimated using similar approaches.

The rumen microbiota had a high prediction accuracy (>73%) to characterize nutrient intake (Table S8). Among CP, NFC and NDF, 31 shared bacteria were observed, and 30 of 31 were predictors identified by the Random Forest classification model. In these shared bacteria, 27 as MRO-associated predictors had a negative correlation associated with the intake of CP, NFC and NDF such as OTU5, OTU24. Considering the other 3 shared features, OTU327 (*Clostridium XlVa*) negatively correlated with the intake of CP, NFC and NDF, while OTU148 (unclassified *Lachnospiraceae*) had no correlation (*p* > 0.05), and OTU6 (*Prevotella*) was positively/moderately correlated (*r* = 0.53, 0.48, 0.49; *p* = 0.023, 0.043, 0.041). Regarding CP and NFC intake, OTU165 (unclassified *Prevotellaceae*) was the shared OTUs abundance. OTU396 (unclassified *Proteobacteria*) and OTU27 (unclassified *Prevotellaceae*) were positively correlated with CP intake (*r* = 0.55, 0.63; *p* = 0.019, 0.005). Observing NDF intake, the abundances of the associated microbiota increased. For example, the OTU464 (unclassified *Burkholderiales*) increased with more NDF intake (*r* = 0.65, *p* = 0.003), while the other OTUs identified as predictors for MCA (i.e., OTU87, OTU83, OTU93, and OTU539) also linearly increased in abundance with an increase of NDF intake (*r* = 0.53, 0.52, 0.49, 0.50 and 0.48) as well. Interestingly, OTU74 (*Treponema*) identified as an MCA signature had no significant association with NDF intake (*r* = 0.38, *p* = 0.124). Moreover, the OTU396 and the core significant *Succiniclasticum* (OTU22) tended to moderately correlate with NDF intake (*r* = 0.46, *p* = 0.051; *r* = 0.41, *p* = 0.09).

Although the regression prediction of MCP and NH_3_-N was not high (50.97% and 44.94%), OTU6 and OTU27 correlating with CP were associated with NH_3_-N, and other regime-associated signatures such as (OTU148) significantly correlated with rumen nitrogen indexes (File S1). Moreover, OTU152, OTU268 and OTU322 had a significant correlation with MCP. The rumen microbiota also provided accurate predictions for VFA concentration (Table S8). Shared OTUs were found in the list between Random Forest classifications and VFA regressions (i.e., 39 acetate, 17 propionate, 24 butyrate, 25 valerate, 36 Total VFA). Those shared OTUs were most of MRO-associated signatures, and negatively correlated with VFA. For the bacteria positively correlating with total VFA, they were observed within one or two of acetate, propionate or butyrate regression models, such as OTU6 within total VFA and butyrate; OTU396 within total VFA and propionate and butyrate. Considering the major VFAs (acetate, propionate and butyrate), OTU83 were the only common microbes correlated positively with (*r* = 0.63, 0.54, 0.56; *p* = 0.005, 0.020, 0.017). When increased acetate was observed, the abundances of OTU122 (*Ruminobacter*), OTU143 (*Fibrobacter*) and OTU204 (unclassified *Bacteroidetes*) tended to increase. Regarding propionate, a positive correlation was found in bacteria, specifically OTU13 (*Prevotella*), OTU93 (*Treponema*), OTU165 (unclassified *Prevotellaceae*), OTU258 (*Olsenella*), OTU120 (*Megasphaera*), OTU532 (unclassified *Bacteroidetes*), OTU322 (*Allisonella*), OTU604 (*Eubacterium*) and OTU530 (*Mitsuokella*). The butyrate-associated bacteria were OTU6, OTU13, OTU539, OTU15 (unclassified *Prevotellaceae*), OTU17 (unclassified *Lachnospiraceae*), OTU114 (*Megasphaera*) and OTU205 (unclassified *Firmicutes*). Notably, a higher ensemble prediction score of 70% in a valerate regression indicated that rumen microbiota were a better predictor as well. When valerate increased, unclassified *Lachnospiraceae* (OTU148 and OTU391), *Olsenella* (OTU20, OTU258), *Megasphaera* (OTU114, OTU120 and OTU173), unclassified *Clostridiales* (OTU311), *Mitsuokella* (OTU152), unclassified *Bacteria* (OTU52), *Prevotella* (OTU186) and unclassified *Porphyromonadaceae* (OTU47) linearly increased.

### 3.5. Rumen Microbiota Driving Function Shifts

To predict how rumen microbiota associate with solid diet supplementary regimes, PICRUSt based on the OTUs’ level was used to predict the abundances of functional categories in the KEGG. In the 3rd level, nutrient pathways were the most popular, presented in Figure S7. Many bacterial genes in all three groups could potentially trigger pathway functions of the same nutrient metabolism, but different treatments participated in different reaction modules. For example, carbohydrate metabolism found in all groups had a specific reaction of pyruvate metabolism and the citrate cycle in MRO; Fructose, mannose Starch and sucrose metabolism in MRC; and glyoxylate and dicarboxylate metabolism in MCA. Moreover, some cellular processes were found in goats supplied with solid diet. MRC contained enriched membrane transport (ABC transporters) and Insulin signaling pathway. Pathways of transcription factors and machinery were found in MRC and MCA.

**Figure 5:**
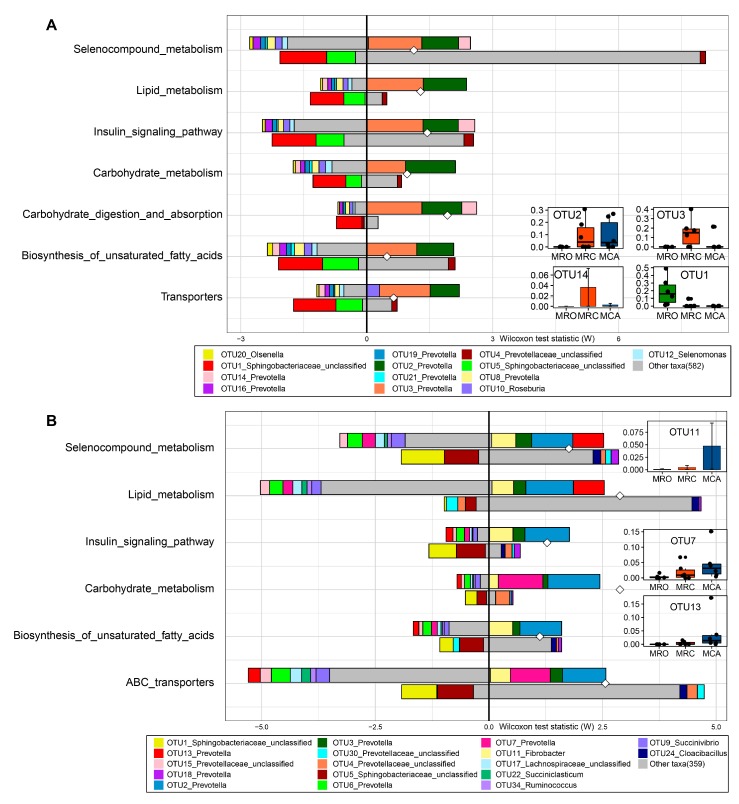
Comparing taxon-level contribution profiles of functional shifts. **A**: The driver OTUs including OTU2, OTU3, OTU14 and OTU1 altered rumen functions when defined MRO as control and MRC as case, **B**: Regarding MRO as control and MCA as case, the OTU11, OTU2, OTU7 and OTU13 drove the rumen functions shifts. Taxon-level shift contribution profiles for case-associated functional modules by Fish Taco. The horizontal axis represents rank and statistic scores, and the vertical axis represents related pathways. For each functional pathway, the bar on the top-right of Y axis represents case-associated bacteria driving the enrichment in the functional module; the bar on the top-left of Y axis indicates case-associated bacteria attenuating functional shift; the bar on the bottom-right of Y axis represents bacteria depleted in control driving functional shift; the bar on the bottom-left of Y axis shows bacteria depleted in control attenuating functional shift. White diamonds represent bacterial-based functional shift scores. The abundances of main drivers were displayed on the right side. OTU2 and OTU3, the shared drivers of enrichments of MRC and MCA were abundant in solid diet regimes. OTU1 enriched in MRO was strongly depleted by solid diets. OTU11, OTU7 and OTU13, driving mainly MCA function shifts, increased abundance with supplementation of solid diet. FishTaco: Functional Shifts’ Taxonomic Contributors; MRO = milk replacer; MRC = milk replacer + concentrate, MCA = milk replacer + concentrate + alfalfa.

Fish Taco was performed to identify the corresponding microbiota driving the functional shifts between supplementary the regimes. There were no differences of normalized abundance of functions between the MRC group as a control and the MCA group as a case based on the Wilcoxon rank-sum test. When the MRO group was used as a control and the case was used as the MRC and MCA groups separately, 31 and 37 significant pathways were found, respectively. Notably, 21 shared functions were also observed between the two comparisons, including metabolism of nutrients (lipid, amino acid, carbohydrate, vitamin, peptidoglycan, terpenoids and polyketides), and the pathway of the endocrine systems cellular processes. (Figure 5 and Figures S8 and S9). To better understand the driver OTUs functions, we identified all sequences with the highest scores contained in the NCBI BALSTN database (File S2). Across all significant functions enriched in the MRC group, a set of *Prevotella* bacteria including OTU3 (*Prevotella copri DSM*), OTU2 (*Prevotella brevis strain GA33*), OTU14 (*Prevotella histicola*) and OTU16 (*Prevotella ruminicola*) (occurrence 100%, 83.3%, 60% and 13.3%) were the main drivers (Figures S8 and S9). While in the MCA group, the function shifts were driven by a convoluted outcome of *Fibrobacter* and *Prevotella* including, OTU11 (*Fibrobacter succinogenes*), OTU2, OTU3, OTU7 (*Prevotella ruminicola*) and OTU13 (*Prevotella brevis strain GA33*) (their occurrence 100%, 100%, 100%, 30.3% and 15.2%). Although the selenocompound metabolism pathway was enriched in MRC and MCA compared to the MRO, the set of OTUs that drove this enrichment of the two feeding regimes as well as the level of contribution to each species differed, with OTU2, OTU3 and OTU14 driving the shift in the MRC group and a set of OTU2, OTU3, OTU11, and OTU13 in the MCA group (Figure 5). These enrichments were attenuated by very different bacteria in the MRC (OTU20, OTU16, OTU8, OTU10, OTU12 and OTU14) and the MCA (OTU15, OTU6, OTU7, OTU17, OTU22 and OTU9). Other highlight pathways such as lipid and carbohydrate metabolism, Biosynthesis of unsaturated fatty acids and transporters had similar patterns. In addition, the MRO enriched microbiota, including unclassified *Sphingobacteriaceae* (OTU1 and OTU5 *Olivibacter sitiensis*) and *Cloacibacillus* (OTU24 *Cloacibacillus porcorum*), were strongly depleted by solid diets.

### 3.6. Network Analysis of Regime Associated Microbiota

**Figure 6:**
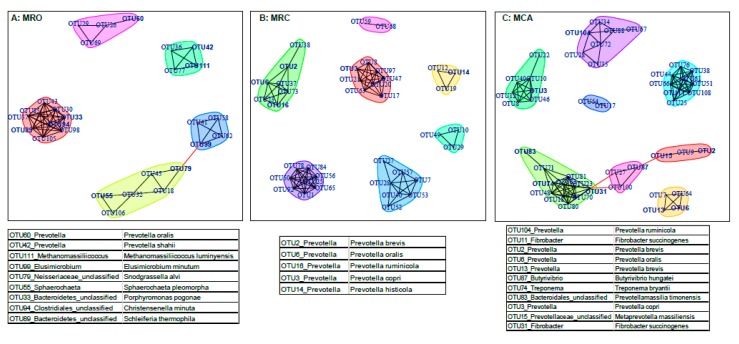
Network analysis of the interactions between bacterial taxa at MRO (**A**), MRC (**B**) and MCA (**C**). The OTUs accounting for >0.5% of the total sequences were selected to network analysis. Each node denotes a particular OTU within the network and each line (edge) represents a significant co-efficiency relationship (Pearson rank correlation coefficient >0.6 or <−0.6). The table under corresponding figures contained the highlight OTUs and their sequence identifiers with the highest scores from NCBI BALSTN database (other OTUs identifiers were shown in File S2). MRO = milk replacer; MRC = milk replacer + concentrate; MCA = milk replacer + concentrate + alfalfa.

Network analysis revealed core sub-community structure within communities that consisted of bacteria associated with the phenotypes and rumen functions in the supplementary regimes. We detected, respectively, four, seven and eight main subnetworks in MRO, MRC and MCA groups (Figure 6). The species that were observed as regimen-associated features and identified as function drivers formed the main subnetwork. In the MRO group, predictors OTU60 (violet cluster), OTU42 and OTU111 (green cluster), OTU99, OTU79, OTU55 (yellow cluster) and OTU33, OTU94, OTU89 (pink cluster) formed the main subnetwork, showing significant correlations with large numbers of other members in the MRO community. For MRC rumen microbiota, OTU2, OTU6 OTU16 in the pale green cluster, OTU3 in the pink cluster and OTU14 in the yellow cluster, were the dominant species associated with other members, which consisted of the main subnetworks. Within the MCA group, OTU104, OTU11, OTU2, OTU6, OTU13, OTU87, OTU74, OTU83 and OTU3 were recognized as main drivers or signatures and were the main members of the respective subnetworks. Their partners’ interaction with these microbiotas may associate with fermentation properties, such as OTU7 and OTU27 in the MCA group. Moreover, OTU79 (*Snodgrassella alvi*) and OTU99 (*Elusimicrobium minutum*) are shown as the hub nodes in the MRO connected yellow and blue cluster, whereas OTU87 (*Butyrivibrio hungatei*), OTU15 (*Metaprevotella massiliensis*) and OTU31 (*Fibrobacter succinogenes subsp. Elongates*) serve as a bridge to link the three clusters.

## 4. Discussion

Early supplementation of a solid diet feeding regimen has shown to have a positive impact on rumen development. A solid diet feeding regimen can influence rumen microbial population and composition, environment alteration and functional achievement. However, the lack of information regarding microbial predictors for supplementary regimes leads to unclear mechanisms involving the manipulation of the rumen microbiota and function shifts. This study confirmed that rumen VFAs, especially acetate, propionate and butyrate, increased significantly with the supplementation of a solid diet, also promoting rumen weight and functions. The predominant genera changed from unclassified *Sphingobacteriaceae* to *Prevotella* when goat kids were supplied a solid diet. The signature microbiota in corresponding feeding regimes significantly correlated with phonotypes such as major nutrient intake and VFA concentration. For example, the biomarkers for MCA (OTU6, OTU87, OTU83, OTU93 and OTU539) were positively correlated with NDF intake and VFA production. The improved rumen function in goats supplied solid diet were shown to be caused by the core bacteria, such as OTU3 (*Prevotella copri DSM*), OTU2 (*Prevotella brevis strain GA33*), OTU14 (*Prevotella histicola*) and OTU11 (*Fibrobacter succinogenes*). These signatures and/or core microbiome formed main sub-communities in response to a solid diet feeding and drive function shifts.

VFAs that are products of the fermentation of diets are shown to be essential to the rumen papillae development and nutrient source for host requirements [23]. In ruminants, VFA produced in the rumen meets 70–80% of the energy requirements for the rumen epithelia, and 50–70% of the energy requirements for the body [24]. In this study, rumen microbial proteins and VFA concentrations increased with the supplementation of solid diet. Other studies have also revealed that early starter and alfalfa consumption facilitated rumen development and changed the pattern of ruminal fermentation [9,11,25]. Moreover, we found that rumen microbial proteins and VFAs were positively correlated with the intake of CP, NDF and NFC. A previous study reported that ruminal NH_3_-N increased linearly in response to increasing dietary CP [26]. This study confirmed that the microbiota in goats fed solid diet had a stronger ability to biosynthesize microbial proteins and VFAs. Additionally, with the exception of the physical stimulation from a solid diet, the chemical effects of nutrient intake could be another reason leading to the increase of VFAs. Therefore, early supplementation of solid diet leading to high nutrient intake that can increase rumen VFA production and nitrogen utilization efficiency reflects that a microbiome experiencing solid diets have a strong ability to utilize nutrients.

In pace with the change of rumen environment, this study also observed that the membership and structure of microbiota also altered when supplied with concentrate or forage compared to only a fluid-fed diet group. Significantly lower alpha diversity in starter-fed-lambs and distinct beta diversity between starter-fed and breast milk-fed lambs was also reported [11]. High bacterial richness in fluid-fed diet groups might be a temporary phenomenon at d60. Others confirmed that rumen microbiota at d70 had a lower richness compared with to d42 [8]. It is known that the rumination phase of the rumen is active after eight weeks, with the transition phase being active between 3–8 weeks. In this study, compared to MRO diets, rumen microbiota in goats supplied with solid diet at d60 may have more mature rumen function and a more stable microbiome structure at the same age. Another reason for the reduction of richness in solid feeding regimes might be due to a high concentration of VFA and a low pH [27]. In addition, the rumen microbiota in solid supplement regimes had similar alpha and beta diversities. The similar pattern could also be observed in rumen fermentation parameters. This might be due to less feed intake of alfalfa and similar concentrate intake. In animal trial, the MCA goats had *ad libitum* access to concentrate and alfalfa pellets in, contained in separate troughs. Based on the feed intake results, goats preferred concentrate. Thus, future studies need to increase roughage intake for examination of its effects on the rumen microbiota, or detect the microbiome after weaned milk replacer.

Random Forest is one of the most popular learning methods commonly used for data exploration [28]. It has been widely used in human microbiome studies to find the signatures for disease or health [29,30]. This study identified important signatures from 838 OTUs using AUCRF, which could provide more effective and accurate information on how diet supplementary regimes affected microbial composition. A higher AUC value (AUC = 1.00) indicates the features are more efficiently classified. Random Forest not only gives an importance score to the significant species, but also finds the accurate bacteria for experimental factors. For example, the low abundances of OTU87, OTU83, OTU93 and OTU539 were identified as the important predictor for regimes, which indicated that low abundance bacteria may also paly critical roles in function drifts. Therefore, previous literature [3,10] only focused on the genera with significantly different abundances and may not provide the best conclusion. Random Forest regression is a useful and robust method for correlation applications because of its ability to automatically produce accuracy estimation and measure the variable importance. Using Random Forest regression to select microbiota with high importance scores would be a corrected method for finding more precise signatures. The percent explained variance is a measure of how well out-of-bag predictions explain the target variance of the training set. High percent explained variance (over 70%) in this study were found in CP, NFC and NDF regression, which indicated that those top microbiota were more important to the responders. Percent explained variance aids in understanding the relationship between specific nutrients and the microbiota. Additionally, using Fish Taco to link the microbiota abundances and rumen function shifts caused by the supplementation of a solid diet regimen is a novel attempt [13]. This technique was used comprehensively integrate the significant species and function shifts. Compared with the original PICRUSt results, there was an improved result of significant pathways. The process relies on a permutation-based approach, a carefully designed normalization, and scaling schemes to preserve overall community taxonomic characteristics and to account for variation induced by each bacteria as well as for variation correlated with community-wide context. Our results identified that a set of core bacteria were the main taxon drivers since low taxonomic abundance profiles were filtered and normalized. Finally, we observed subnetworks formed by these signature microbiota and their partners. In summary, greater algorithms gave insights as to how microbiota had impacts on rumen function shifts.

Rumen microbiota degrade fibers, polysaccharides and proteins in the diet and yield VFAs and microbial proteins, which offer nutrients to meet the host’s requirement for maintenance and growth [31,32]. Based on the mentioned machine leaning algorithms, we analyzed the microbiome to link supplementary regime to the alteration of the rumen environment and function. Supplemental solid diets altered the core microbiota from unclassified *Sphingobacteriaceae* to *Prevotella*. The representative OTU5 associated with unclassified *Sphingobacteriaceae* as the MRO group predictors were negatively associated with macronutrient intake and VFA production. OTU5 classified as *Olivibacter sitiensis* function is not yet clear, but it decreased with pH reduction when there was an intake of high concentrate diet [33]. The species affiliated with *Prevotella* (OTU2, OTU3, OTU6 and OTU13) in the top 30 increased in solid supplementary regimes. Other studies also reported that the abundances of the genus *Prevotella* that were predominant in starter-fed lambs positively correlated with acetate, propionate and urea nitrogen concentration [3,8,11]. This genus is efficient at utilizing proteins and carbohydrates (either fiber- or non-fiber-carbohydrate) [34]. Notably, OTU6 (*Prevotella oralis*) was identified as a signature species for solid diets, correlating with macronutrient intake and butyrate concentration. The microbiota OTU13 (*Prevotella brevis strain GA33*) was not classified as a predictor for the MCA group, but we observed a high abundance in the MCA group, positively associating with propionate and butyrate, driving function shifts and interactions with the other core microbiome. Therefore, increased abundance of these two species represented as *Prevotella* in the rumen accessed solid diets promoted the improvement of rumen digestibility and function by yielding greater VFA products. OTU2 (*Prevotella brevis*) and OTU3 (*Prevotella copri*) were the main drivers for function shifts by solid diet. De Filippis et al. detected distinct strains of *Prevotella copri* by metagenome studies and showed that fiber-rich diets were linked to these strains with improved potential for carbohydrate catabolism [35]. Broadly, introduction of a solid fiber-rich diet to goats before weaning demonstrated a proliferation of *Prevotella*, which was also observed in other large domestic animals [36]. This reveals they could be used as potential microbiota to utilize a solid diet, as well as maintain rumen community balance and prevent metabolic disease caused by dysbiosis. Another core genus increased in both solid diet regimes was unclassified *Lachnospiraceae*. The family *Lachnospiraceae* contains many known plant degrading species and most of the butyrate-producers [37]. In our results, OTU148-*Lachnospiraceae* (*Kineothrix alysoides*) enriched in the MRC group was significantly associated with NH_3_-N and valerate, although it was identified by a regression model for nutrient intake and fermentation parameters. Regarding other dominant bacteria, *Roseburia* and *Selenomonas* specifically increased in concentrate diet regime. The abundances of *Fibrobacter*, *Treponema* and *Succinivibrio* arose in the excess supplementation of alfalfa. Another microbial known as *Succinivibrio*, a saccharolytic bacteria, has been shown to yield acetate and lactate [38]. The OTUs associated with these genera were not observed well in our study. For example, OTU10-*Roseburia* and OTU9-*Succinivibrio* formed main structures with other members; OTU74 affiliated with *Treponema* predicted the MCA group well except in relation to phonotypes; and OTU11-*Fibrobacter* drove the enriched functions of MCA while OTU143-*Fibrobacter* increased with acetate. These microbes at either the genus or OTUs level had significant abundances in different regimes, however, they were not correlated with phonotypes well. Reasons could be that they were symbiotic with other microbiota. For example, *Treponema* does not utilize fiber, but it helps other bacteria to digest cellulosic materials [39].

The MCA-associated features, OTU87 (*Butyrivibrio hungatei*), OTU83 (*Prevotellamassilia timonensis*), OTU539 (*Abyssivirga alkaniphila*), and OUT93 (*Treponema pectinovorum*), correlated positively with NDF intake. Nevertheless, OTU539 associated with butyrate production and OTU93 related with propionate, while OTU83 correlated with all three major VFAs. *Butyrivibrio hungatei* is the primary butyrate producer in the rumen and effectively degrades hemicellulose [40]. *Prevotellamassilia timonensis* is a hemicellulose-degrading bacteria [41]. *Abyssivirga alkaniphila* ferments saccharides, peptides and amino acids [42]. *Fibrobacter* and *Treponema* synergistically break down the fiber components [43,44]. The function of OTU396 (*Pelobacter propionicus*) and OTU165 (*Marseilla massiliensis*) is hypothesized to be similar with OTU6. We observed their association with macronutrient and major VFA analyses. MCA signatures cooperatively digest carbohydrate or protein and produce VFAs. An increase of butyrate is as an important regulatory factor and a stimulator of rumen development, and was demonstrated [45] with the supplementation of alfalfa (NDF), which could improve rumen development by increasing abundances of these synergistic bacteria. In addition, the OTU27 (*Prevotella falsenii*) is hypothesized to be a nitrogen-associated bacteria since it correlated with CP intake and NH_3_-N effectively, though it was not classified as MCA signatures. A review reported some strains in *Prevotella* can degrade dietary proteins [39]. Therefore, those signatures for regimes supplied with alfalfa contribute to both protein and carbohydrate utilization and yield more nitrogen materials and VFAs for host development. By contrast, the MRO signature microbiota cannot promote rumen functions at the ruminant phase, however, they still may provide some baseline information of the rumen in the non-ruminant stage. Except *Sphingobacteriaceae*, OTU24 (*Cloacibacillus*) was another important specie for goats fed only milk replacer as a nutrient source. *Cloacibacillus* is a novel bacterium that degrades amino acids and produces VFAs [46]. These MRO-associated signatures could be considered as passengers that contribute to rumen development at a specific time. Although little is known about the contribution of these bacteria, they are important for digestion in milk replacer and could be the primary strains impacted on late bacterial colonization.

The first limitation of this study is small sample size (6 per groups). However, it still showed good results between fluid diet and supplement of solid diet, providing insights for future large-scale studies. Secondly, alfalfa in the MCA groups was provided *ad libitum*, resulting in less intake and less significant effects compared to adding alfalfa, but many bacteria related with fiber digestion were still observed due to significant fiber effects. Moreover, most of the MRO group signature functions were not well described and are required for the identification of their functions by longitudinal measurements in further studies. Finally, the accuracy of functional predictions of rumen microbiota may be reduced because the original PICRUSt is based on microbial genomes from the human mirobiome [47]. In the future, metagenomic and/or metabolomic analysis for rumen microbiota should be performed. Despite these limitations, we confirmed that signature microbiota for supplementary solid diet plays important roles in the promotion of rumen functions.

## 5. Conclusions

Rumen fermentation and microbial composition were altered by the supplementation of concentrate or concentrate plus alfalfa, particularly the latter, in the early life of goat kids. The concentration of rumen VFAs, especially acetate, propionate and butyrate increased significantly when goats intake more nutrients from solid diet, and positively correlated with intake of crude protein, non-fiber carbohydrate and neutral detergent fiber. The membership and structure of rumen microbiota were altered. This study also identified a set of signatures for supplementary solid diet regimes and validated their association with macronutrient intake and rumen fermentation. It is notable that this is the first time to use Fish Taco in the determination of a link between those signatures’ abundances and rumen function shifts. We additionally performed network analysis to detect the interaction of signatures. By comprehensive integration, many members of bacteria that have symbiotic relationships with signatures were classified. Therefore, in goat kids, extra nutrients from concentrate and/or forage manipulated the core community structures by significantly altering specific signature microbiota and their symbiotic partners, and furthermore increased volatile fatty acids were produced, eventually promoting rumen development and functions. Our study answers several important questions in rumen microbiome affected by a supplementary solid diet, and offers a foundation for studies aimed at improving ruminant health and production.

## Figures and Tables

**Table 1:** Correlation analysis between nutrient (CP, NFC and NDF) intake and rumen microbes in goat kids. We performed the Random Forest regression model across all samples between dietary average daily CP, NFC and NDF intake and all the bacteria with high prediction accuracy (Table S6). Then, using the abundances of top 50 features to calculate the Pearson correlation with intake of CP, NFC and NDF was carried out. We consider *p* < 0.05 as a significant correlation. The bacteria from up to bottom followed the descending order of mean square error. CP: Crude protein average daily intake; NDF: Neutral detergent fibers average daily intake; NFC: Non-fibrous carbohydrates average daily intake.

CP	*r*	*p* Value
OTU165 *Prevotellaceae*_unclassified	0.48	0.04
OTU24 *Cloacibacillus*	−0.7	0
OTU313 *Methanomassiliicoccus*	−0.65	0
OTU154 *Methanomassiliicoccus*	−0.84	0
OTU39 *Ruminococcaceae*_unclassified	−0.86	0
OTU60 *Prevotella*	−0.79	0
OTU111 *Methanomassiliicoccus*	−0.58	0.01
OTU119 *Prevotella*	−0.6	0.01
OTU365 *Neisseriaceae*_unclassified	−0.8	0
OTU5 *Sphingobacteriaceae*_unclassified	−0.54	0.02
OTU79 *Neisseriaceae*_unclassified	−0.7	0
OTU99 *Elusimicrobium*	−0.52	0.03
OTU90 *Bacteroidetes*_unclassified	−0.73	0
OTU55 *Sphaerochaeta*	−0.56	0.02
OTU396 *Proteobacteria*_unclassified	0.55	0.02
OTU290 *Bacteroidetes*_unclassified	−0.52	0.03
OTU132 *Pyramidobacter*	−0.61	0.01
OTU296 *Clostridiales*_unclassified	−0.74	0
OTU330 *Firmicutes*_unclassified	−0.7	0
OTU169 *Bacteroidetes*_unclassified	−0.68	0
OTU6 *Prevotella*	0.53	0.02
OTU89 *Bacteroidetes*_unclassified	−0.38	0.12
OTU168 *Methanomicrobium*	−0.56	0.02
OTU298 *Pyramidobacter*	−0.6	0.01
OTU279 *Clostridiales*_unclassified	−0.61	0.01
OTU422 *Porphyromonadaceae*_unclassified	−0.56	0.02
OTU506 *Bacteria*_unclassified	−0.75	0
OTU387 *Bacteroidetes*_unclassified	−0.78	0
OTU548 *Bifidobacterium*	−0.6	0.01
OTU327 *Clostridium.XlVa*	−0.65	0
OTU217 *Bacteroidetes*_unclassified	−0.53	0.02
OTU75 *Ruminococcaceae*_unclassified	−0.88	0
OTU385 *Moraxella*	−0.58	0.01
OTU94 *Clostridiales*_unclassified	−0.52	0.03
OTU178 *Bacteroides*	−0.4	0.1
OTU411 *Clostridia*_unclassified	−0.8	0
OTU139 *Bacteroidetes*_unclassified	−0.61	0.01
OTU245 *Clostridiales*_unclassified	−0.76	0
OTU147 *Bacteroidales*_unclassified	−0.55	0.02
OTU487 *Firmicutes*_unclassified	−0.82	0
OTU287 *Pasteurellaceae*_unclassified	−0.71	0
OTU13 *Prevotella*	0.42	0.09
OTU219 *Bacteroidetes_*unclassified	−0.52	0.03
OTU392 *Bilophila*	−0.63	0
OTU208 *Bacteria*_unclassified	−0.56	0.01
OTU281 *Bacteroidetes*_unclassified	−0.7	0
OTU266 *Porphyromonadaceae*_unclassified	−0.73	0
OTU151 *Ruminococcaceae*_unclassified	−0.65	0
OTU27 *Prevotellaceae*_unclassified	0.63	0.01
OTU148 *Lachnospiraceae*_unclassified	0.19	0.45
**NFC**	***r***	***p*** **Value**
OTU165 *Prevotellaceae*_unclassified	0.41	0.09
OTU365 *Neisseriaceae*_unclassified	−0.84	0
OTU39 *Ruminococcaceae*_unclassified	−0.91	0
OTU154 *Methanomassiliicoccus*	−0.87	0
OTU296 *Clostridiales*_unclassified	−0.78	0
OTU5 *Sphingobacteriaceae*_unclassified	−0.55	0.02
OTU111 *Methanomassiliicoccus*	−0.6	0.01
OTU60 *Prevotella*	−0.82	0
OTU24 *Cloacibacillus*	−0.7	0
OTU6 *Prevotella*	0.49	0.04
OTU313 *Methanomassiliicoccus*	−0.67	0
OTU119 *Prevotella*	−0.64	0
OTU55 *Sphaerochaeta*	−0.56	0.01
OTU79 *Neisseriaceae*_unclassified	−0.73	0
OTU222 *Firmicutes*_unclassified	−0.67	0
OTU114 *Megasphaera*	0.36	0.14
OTU94 *Clostridiales*_unclassified	−0.54	0.02
OTU90 *Bacteroidetes*_unclassified	−0.75	0
OTU279 *Clostridiales*_unclassified	−0.64	0
OTU290 *Bacteroidetes*_unclassified	−0.54	0.02
OTU178 *Bacteroides*	−0.41	0.09
OTU385 *Moraxella*	−0.6	0.01
OTU281 *Bacteroidetes*_unclassified	−0.72	0
OTU266 *Porphyromonadaceae*_unclassified	0.77	0
OTU217 *Bacteroidetes*_unclassified	−0.57	0.01
OTU62 *Prevotellaceae_*unclassified	−0.46	0.06
OTU148 *Lachnospiraceae*_unclassified	0.27	0.28
OTU33 *Bacteroidetes*_unclassified	−0.51	0.03
OTU75 *Ruminococcaceae*_unclassified	−0.91	0
OTU99 *Elusimicrobium*	−0.55	0.02
OTU192 *Methanimicrococcus*	−0.77	0
OTU89 *Bacteroidetes*_unclassified	−0.39	0.11
OTU487 *Firmicutes*_unclassified	−0.86	0
OTU208 *Bacteria*_unclassified	−0.61	0.01
OTU330 *Firmicutes*_unclassified	−0.72	0
OTU422 *Porphyromonadaceae*_unclassified	−0.56	0.02
OTU477 *Ruminococcaceae*_unclassified	−0.81	0
OTU182 *Bacteroidetes_*unclassified	−0.67	0
OTU190 *Sphaerochaeta*	−0.71	0
OTU387 *Bacteroidetes*_unclassified	−0.8	0
OTU147 *Bacteroidales*_unclassified	−0.57	0.01
OTU506 *Bacteria*_unclassified	−0.76	0
OTU139 *Bacteroidetes*_unclassified	−0.63	0
OTU413 *Clostridium.XlVb*	−0.77	0
OTU270 *Bibersteinia*	−0.68	0
OTU392 *Bilophila*	−0.67	0
OTU411 *Clostridia*_unclassified	−0.81	0
OTU245 *Clostridiales*_unclassified	-0.8	0
OTU327 *Clostridium.XlVa*	−0.69	0
OTU255 *Comamonas*	−0.52	0.03
**NDF**	***r***	***p*** **Value**
OTU39 *Ruminococcaceae*_unclassified	−0.9	0
OTU313 *Methanomassiliicoccus*	−0.66	0
OTU365 *Neisseriaceae*_unclassified	−0.84	0
OTU6 *Prevotella*	0.48	0.04
OTU5 *Sphingobacteriaceae*_unclassified	−0.54	0.02
OTU148 *Lachnospiraceae*_unclassified	0.27	0.28
OTU119 *Prevotella*	−0.65	0
OTU60 *Prevotella*	−0.82	0
OTU114 *Megasphaera*	0.36	0.14
OTU154 *Methanomassiliicoccus*	−0.85	0
OTU539 *Clostridiales*_unclassified	0.49	0.04
OTU24 *Cloacibacillus*	−0.68	0
OTU296 *Clostridiales*_unclassified	−0.78	0
OTU111 *Methanomassiliicoccus*	−0.59	0.01
OTU55 *Sphaerochaeta*	−0.55	0.02
OTU104 *Prevotella*	0.29	0.24
OTU79 *Neisseriaceae*_unclassified	−0.73	0
OTU87 *Butyrivibrio*	0.5	0.03
OTU396 *Proteobacteria*_unclassified	0.46	0.05
OTU93 *Treponema*	0.53	0.03
OTU270 *Bibersteinia*	−0.67	0
OTU132 *Pyramidobacter*	-0.67	0
OTU298 *Pyramidobacter*	−0.59	0.01
OTU99 *Elusimicrobium*	−0.54	0.02
OTU90 *Bacteroidetes*_unclassified	−0.73	0
OTU57 *Prevotella*	0.18	0.46
OTU281 *Bacteroidetes_*unclassified	−0.71	0
OTU94 *Clostridiales_*unclassified	−0.53	0.02
OTU139 *Bacteroidetes*_unclassified	−0.62	0.01
OTU83 *Bacteroidales*_unclassified	0.52	0.03
OTU287 *Pasteurellaceae*_unclassified	−0.73	0
OTU89 *Bacteroidetes*_unclassified	−0.39	0.11
OTU74 *Treponema*	0.38	0.12
OTU330 *Firmicutes*_unclassified	−0.71	0
OTU290 *Bacteroidetes*_unclassified	−0.54	0.02
OTU13 *Prevotella*	0.33	0.17
OTU22 *Succiniclasticum*	0.41	0.09
OTU392 *Bilophila*	−0.67	0
OTU190 *Sphaerochaeta*	−0.71	0
OTU208 *Bacteria*_unclassified	−0.62	0.01
OTU327 *Clostridium.XlVa*	−0.69	0
OTU464 *Burkholderiales*_unclassified	0.65	0
OTU245 *Clostridiales*_unclassified	−0.8	0
OTU147 *Bacteroidales*_unclassified	−0.56	0.02
OTU506 *Bacteria*_unclassified	−0.73	0
OTU169 *Bacteroidetes*_unclassified	−0.72	0
OTU178 *Bacteroides*	−0.39	0.11
OTU75 *Ruminococcaceae*_unclassified	−0.89	0
OTU487 *Firmicutes*_unclassified	−0.85	0
OTU192 *Methanimicrococcus*	−0.77	0

## Supplementary Materials

The following are available online at https://www.mdpi.com/2076-2607/7/11/516/s1.
