# Supplementary material for: The Signature Microbiota Drive Rumen Function Shifts in Goat Kids Introduced to Solid Diet Regimes
Source: Microorganisms. 2019 Oct 31;7(11):516. doi: 10.3390/microorganisms7110516 (PMC6921049; doi:10.3390/microorganisms7110516)
Supplement: Supplementary file 1 [file microorganisms-07-00516-s001.zip › microorganisms-597628-supplementary -for conversion/microorganisms-597628-supplementary-for conversion.pdf]

Supplementary Information

The Signature Microbiota Drive Rumen Function Shifts in Goat Kids Introduced to Solid Diet Regimes

Xiaokang Lv [1,†], Jianmin Chai [1,2,†], Qiyu Diao[1], Wenqin Huang[1], Yimin Zhuang[1], Naifeng Zhang [1,*]

[1] Feed Research Institute, Chinese Academy of Agricultural Sciences, Key Laboratory of Feed Biotechnology of the Ministry of Agriculture, Beijing, 100081, China; 13121191399@163.com (X.K.L.); jchai@uark.edu (J.M.C.); diaoqiyu@caas.cn (Q.D.)； m13121271017@163.com (W.H.); zym1164323345@163.com (Y.Z.)

[2] Department of Animal Science, Division of Agriculture, University of Arkansas, Fayetteville, AR, 72701, USA

* Correspondence: zhangnaifeng@caas.cn Postal address: No.12 Zhongguancun South St., Haidian District, Beijing, P.R.China 100081, +86(10) 8210 6055

† These authors contributed equally to this work.

Materials and Methods

Goat Kid Management

Goat kids remained with their mother and received breast milk from 0 to 20 days. During 20 to 60 days of age, they were separated with their dams and the above three kinds of diets were provided to corresponding groups. Other management strategies including vaccination, cleaning and disinfection of pens followed farm normal policy. All animals were fed with milk replacer from 20 to 60 days. Feeding amount of milk replacer were 2% body weight. Goat kids were fed four times a day (0600, 1200, 1800 and 2200) at 20–30 days, and thrice daily at 30–60 days (0600, 1200 and 1800). The milk replacer was dissolved with hot water cooled to 65–70 °C after boiling, and offered to goat kids when it was cooled to 40 ± 1 °C. The ratio of milk replacer to water was 1:6 (weight (g)/ volume (ml)). The milk replacer (China Patent product ZL02128844.5) used in the experiment was provided by Beijing Precision Animal Nutrition Research Center. The concentrate with ingredients of corn, soybean etc. was purchased from Cargill Feed Company (Nanjing, China). The alfalfa pellets purchased from Baofa Agriculture and Animal Husbandry Co. Ltd, (Gansu, China) had the same diameter (4 mm) as the diet concentrate. During the animal trial, all the goat kids had *ad libitum* access to water, the MRC and MCA kids were freely to access concentrate, and the MCA goats had extra free choice of alfalfa pellets. The nutritional levels of milk replacer, concentrate and alfalfa pellets are shown in Table S1.

Sample Collection and Chemical Analysis

Daily feed intakes were recorded in animal trial. Feed samples were collected, dried in a forced-air oven at 65 °C for 48 h and analyzed for crude protein (CP), non-fiber carbohydrate (NFC), and neutral detergent fiber (NDF) according to the Association of Official Analytical Chemists [1]. Then, average daily intake of CP, NFC and NDF were calculated. Only data of table S1 (dietary composition) and table S2 (growth performance) were published in a Chinese journal paper [2], and other data, such as rumen fermentation parameters and microbiome analysis, are detailed in this manuscript.

Data Analysis

Rumen fermentation parameters were shown using bar charts made in R (v3.6.0) by 'ggplot2' package. The ANOVA test was used for significance calculation after detection of homogeneity of variance. After the global test was significant, a post-hoc analysis (Tukey's HSD test) was performed to determine which

group of the independent variable differ from each other group. The remaining fermentation parameters were shown in Table S3.

The Pearson correlation was performed for measurements of association between nutrient intake with rumen fermentation parameters (shown in Table S4), and between phenotypes and alpha diversity (table S5). To better understand the rumen microbial composition in goats introduced different solid diet regime, stacked bar charts of the top 30 genus were made (Figure S1), and significant OTUs were shown in table S6. The p values were calculated by using Kruskal–Wallis test and a post-hoc Dunn Kruskal-Wallis multiple comparison with Bonferroni adjustment to evaluate differences between two groups in R.

A Random Forest classification model was performed to identify the top microbiome signatures to differentiate three supplementary feeding regimes. R package 'AUCRF' (v.1.1) was used to process Random Forest model and select optimal variables based on the area-under-the receiver operator characteristic curve (AUC) of the Random Forest method (AUCRF) [3]. The relative abundances of all the microbiota were included for predictors' selection. The 'ntree' parameters was set at 10,000 in the model. For calculation the probability of each selected variable, a 10-fold cross validation analysis and 20 times repetitions of cross validation were performed. The model accuracy, including AUC, sensitivity and specificity of variables, was calculated using the 'pROC' package (v.1.13). Thus, variables importance plot was generated based on the importance scores (Mean Decrease in Accuracy, MDA) of optimal features and their boxplots of selected features were drawn in R. Firstly, all 3 groups were analyzed together, and then pair wise comparison. The AUC (area under the curve), specificity, and sensitivity were showed in (Figure S2). The top predictors identified by AUCRF model were displayed in (Table S7), and their abundances were showed in Figure S3-S6.

The Random Forest regression model was used to select the rumen microbiota that were important for average daily intake of major nutrients (i.e., CP, NDF and NFC) and rumen fermentation parameters. The model was run in R software using 'RandomForest' package (v 4.6-14). The percent variance explained was reported for the estimation of accuracy of regression model. The top 50 selected features were then analyzed Pearson correlation with those macro indicators respectively. All the Pearson regression results were in File S1.

Predictive function analysis was performed using the PICRUSt algorithm based on the Kyoto Encyclopedia of Genes and Genomes (KEGG) classification using the closed-reference OTUs [4]. The level 3 of functional prediction was shown in Figure S7. The Functional Shifts' Taxonomic Contributors (Fish Taco) software was used to find the rumen bacteria driving the functional shifts between supplementary regimes in this study. The Fish Taco software running on Python system followed the official pipeline at http://borenstein-lab.github.io/fishtaco/execution.html. A taxonomic abundance at OTUs' level and functional abundance profile at levels 3 from the PICRUSt analysis were used. The primary statistical results were presented in Files S2-S3. In pairwise comparisons, we labeled MRO groups as control and MRC or MCA as case, and tested MRC as control vs MCA as case. Each functional shift was grouped into case-associated with driving case-enrichment or attenuating case-enrichment, and control-associated driving case-enrichment or attenuating case-enrichment. The output results visualization was performed in FishTacoPlot package in R (Version 3.6.0). Except the representative and significant pathway showed in main text, others were disclosed in Figures S8-S9.

Table S1. Nutritional components of milk replacer, starter, and alfalfa pellets.

Items	Milk Replacer	Starter Pellets	Alfalfa Pellets
Chemical composition, % of DM basis			
DM (%)	95.50	95.25	95.53
Total Energy (MJ/Kg)	20.74	18.03	17.39
CP (%)	25.52	19.63	14.77
EE (%)	15.96	3.40	1.04
NDF (%)	---	29.62	51.45
ADF (%)	---	8.64	35.20
Ash (%)	6.54	7.99	11.98
Ca (%)	1.02	0.95	0.90
P (%)	0.66	0.70	0.48

DM:Dry matter; CP:Crude protein; EE: Ether extract; NDF: Neutral detergent fibers; ADF: Acid detergent fiber; Ash: Crude ash.

Table S2. Effect of early supplementary solid diet on growth performance of goat kids

Items	Treatments			SEM	*p*-value
	MRO	MRC	MCA		
Milk replacer intake/(g•d^{-1})	130.62	107.91	119.90	4.95	0.1696
Concentrate intake/(g•d^{-1})	---	188.18	189.56	4.82	0.8956
Alfalfa pellets intake/(g•d^{-1})	---	---	34.34	---	---
Dry matter intake/(g•d^{-1})	122.47 ^{c}	271.50 ^{b}	309.97 ^{a}	16.12	<0.0001
Protein intake/(g•d^{-1})	31.33 ^{c}	58.52 ^{b}	66.37 ^{a}	4.07	<0.0001
NDF-Neutral detergent fiber intake/(g•d^{-1})	---	49.25 ^{b}	66.08 ^{a}	3.13	0.0004
NFC-Non-fibrous carbohydrate intake/(g•d^{-1})	63.82 ^{c}	118.16 ^{b}	131.15 ^{a}	7.94	<0.0001

MRO = milk replacer, MRC= milk replacer + concentrate, MCA= milk replacer + concentrate + alfalfa; SEM = Standard error of the means. Values with different small letter superscripts in the same row mean significant difference ($p < 0.05$).

Only the data of Table S1 (dietary composition) and Table S2 (growth performance) were published in a Chinese journal article (doi 10.11843/j.issn.0366-6964.2019.05.011), thus the authors have translated them into English since the nutrient intake were used in this draft.

Table S3. Effects of early supplementary solid diet on growth performance and rumen fermentation parameters in goat kids

Items	Treatments[1]			SEM	*p*-value
	MRO	MRC	MCA		
Slaughter BW, kg	7.01 ^{b}	10.47 ^{a}	10.23 ^{a}	0.41	<0.000 1
Rumen weight, g	47.42^{c}	126.70 ^{b}	175.67^{a}	14.94	<0.001
Average daily gain/g	70.28 ^{b}	123.93 ^{a}	126.25^{a}	5.48	<0.0001
pH	7.09 ^{a}	5.88 ^{b}	6.01^{b}	0.15	<0.0001
Isobutyrate, mmol/L	0.24	0.48	0.23	0.08	0.3589
Isovalerate, mmol/L	0.56	0.98	0.91	0.16	0.5470
Valerate, mmol/L	0.73 ^{b}	7.34 ^{a}	5.01^{a}	0.95	0.0045
A:P	2.55	2.23	2.20	0.13	0.5502

MRO = milk replacer, MRC = milk replacer + concentrate, MCA = milk replacer + concentrate + alfalfa; SEM = Standard error of the means. BW=body weight; A:P = the ratio of acetate and propionate. Values with different small letter superscripts in the same row mean significant difference ($p < 0.05$) .

Table S4. Pearson correlation between nutrient and rumen fermentation

Items	CP_ADI		NDF_ADI		NFC_ADI		Slaughter body weight	
	coefficient	p-value	coefficient	p-value	coefficient	p-value	coefficient	p-value
pH	−0.83	0.000	−0.85	0.000	−0.86	0.000	−0.78	0.000
NH_3-N	−0.78	0.000	−0.82	0.000	−0.82	0.000	−0.76	0.000
MCP	0.80	0.000	0.88	0.000	0.86	0.000	0.82	0.000
acetate	0.92	0.000	0.94	0.000	0.94	0.000	0.78	0.000
propionate	0.82	0.000	0.82	0.000	0.82	0.000	0.67	0.002
butyrate	0.87	0.000	0.88	0.000	0.87	0.000	0.86	0.000
valerate	0.60	0.009	0.65	0.004	0.66	0.003	0.76	0.000
Total VFA	0.94	0.000	0.96	0.000	0.96	0.000	0.86	0.000

CP_ADI: Crude protein average daily intake; NDF_ADI: Neutral detergent fibers average daily intake; NFC_ADI: Non-fibrous carbohydrates average daily intake; Total VFA: Total volatile fatty acids; MCP: Microbial crude protein. A *p* value equal 0 means it was smaller than 0.0005.

Table S5. Pearson correlation analysis between phenotypes and Alpha diversity in goat kids.

Items	Shannon Index		Observed species		Chao1	
	coefficients	p-value	coefficients	p-value	coefficients	*p*-value
CP	−0.04	0.880	−0.65	0.003	−0.66	0.003
NFC	−0.12	0.642	−0.73	0.001	−0.70	0.001
NDF	−0.13	0.594	−0.74	0.001	−0.69	0.001
acetate	−0.09	0.717	−0.64	0.004	−0.59	0.011
propionate	−0.10	0.687	−0.58	0.011	−0.57	0.014
butyrate	−0.15	0.557	−0.71	0.001	−0.72	0.001
Total VFA	−0.18	0.484	−0.75	0.000	−0.71	0.001
valerate	−0.58	0.012	−0.88	0.000	−0.78	0.000
pH	0.44	0.066	0.81	0.000	0.76	0.000
NH_3-N	0.15	0.562	0.63	0.005	0.63	0.005
MCP	−0.41	0.092	−0.79	0.000	−0.66	0.003
Slaughter body weight	−0.16	0.534	−0.76	0.000	−0.74	0.000

CP_ADI: Crude protein average daily intake; NDF_ADI: Neutral detergent fibers average daily intake; NFC_ADI: Non-fibrous carbohydrates average daily intake; Total VFA: Total volatile fatty acids; MCP: Microbial crude protein. The *p* value equal to 0 means it was smaller than 0.0005.

Table S6 the average abundance of 281 significant OTUs

OTUID	MRO	MRC	MCA	*p*-value-KWtest
OTU1_*Sphingobacteriaceae*_unclassified	0.19182	0.01601	0.00000	0.0013
OTU2_*Prevotella*	0.00041	0.09529	0.09803	0.0401
OTU4_*Prevotellaceae*_unclassified	0.11709	0.00032	0.00036	0.0418
OTU5_*Sphingobacteriaceae*_unclassified	0.11134	0.00036	0.00002	0.0020
OTU6_*Prevotella*	0.00005	0.01349	0.05890	0.0022
OTU9_*Succinivibrio*	0.00036	0.01498	0.04738	0.0395
OTU10_*Roseburia*	0.00003	0.04486	0.00896	0.0264
OTU13_*Prevotella*	0.00001	0.00414	0.04023	0.0037
OTU15_*Prevotellaceae*_unclassified	0.00000	0.00291	0.03540	0.0026
OTU18_*Prevotella*	0.01016	0.00016	0.01923	0.0168
OTU20_*Olsenella*	0.00010	0.02279	0.00472	0.0192
OTU21_*Prevotella*	0.00007	0.02081	0.00291	0.0392
OTU22_*Succiniclasticum*	0.00000	0.00491	0.01817	0.0060
OTU24_*Cloacibacillus*	0.02164	0.00001	0.00000	0.0008
OTU27_*Prevotellaceae*_unclassified	0.00001	0.00756	0.01181	0.0307
OTU30_*Prevotellaceae*_unclassified	0.01838	0.00035	0.00000	0.0287
OTU33_*Bacteroidetes*_unclassified	0.01775	0.00000	0.00000	0.0003
OTU34_*Ruminococcus*	0.00000	0.00262	0.01489	0.0057
OTU36_*Candidatus.Endomicrobium*_unclassified	0.01322	0.00298	0.00000	0.0034
OTU38_*Prevotellaceae*_unclassified	0.00001	0.00982	0.00576	0.0281
OTU39_*Ruminococcaceae*_unclassified	0.01473	0.00020	0.00024	0.0026
OTU40_*Bacteroidetes*_unclassified	0.00000	0.00663	0.00808	0.0079
OTU43_*Bacteroidetes*_unclassified	0.01254	0.00000	0.00000	0.0345
OTU44_*Prevotellaceae*_unclassified	0.00000	0.00001	0.01248	0.0249
OTU47_*Porphyromonadaceae*_unclassified	0.00007	0.00952	0.00262	0.0395
OTU52_*Bacteria*_unclassified	0.00002	0.01040	0.00119	0.0189
OTU53_*Lachnospiraceae*_unclassified	0.00000	0.01090	0.00021	0.0486
OTU54_*Prevotella*	0.00000	0.00000	0.01110	0.0085
OTU55_*Sphaerochaeta*	0.01053	0.00006	0.00001	0.0011
OTU57_*Prevotella*	0.00000	0.00774	0.00267	0.0092
OTU58_*Bacteroidetes*_unclassified	0.01022	0.00000	0.00000	0.0085
OTU60_*Prevotella*	0.00828	0.00119	0.00047	0.0030
OTU62_*Prevotellaceae*_unclassified	0.00955	0.00000	0.00008	0.0013
OTU63_*Prevotella*	0.00003	0.00682	0.00276	0.0366
OTU67_*Treponema*	0.00000	0.00000	0.00906	0.0085
OTU71_*Lachnospiraceae*_unclassified	0.00000	0.00020	0.00833	0.0287
OTU74_*Treponema*	0.00001	0.00000	0.00818	0.0055
OTU75_*Ruminococcaceae*_unclassified	0.00818	0.00000	0.00000	0.0003
OTU77_*Bacteroidetes*_unclassified	0.00812	0.00000	0.00000	0.0085
OTU79_*Neisseriaceae*_unclassified	0.00744	0.00013	0.00031	0.0042
OTU83_*Bacteroidales*_unclassified	0.00005	0.00026	0.00727	0.0053
OTU86_*Pyramidobacter*	0.00000	0.00354	0.00330	0.0053
OTU87_*Butyrivibrio*	0.00023	0.00001	0.00657	0.0048
OTU89_*Bacteroidetes*_unclassified	0.00668	0.00000	0.00000	0.0003
OTU90_*Bacteroidetes*_unclassified	0.00664	0.00001	0.00000	0.0006
OTU93_*Treponema*	0.00013	0.00000	0.00646	0.0028
OTU94_*Clostridiales*_unclassified	0.00646	0.00000	0.00000	0.0003
OTU97_*Clostridiales*_unclassified	0.00001	0.00515	0.00097	0.0422

Table S6. *Cont.*

OTU98_*Anaeroplasma*	0.00597	0.00000	0.00000	0.0345
OTU99_*Elusimicrobium*	0.00596	0.00001	0.00000	0.0006
OTU100_*Prevotellaceae*_unclassified	0.00001	0.00025	0.00568	0.0161
OTU103_*Prevotellaceae*_unclassified	0.00479	0.00006	0.00076	0.0448
OTU104_*Prevotella*	0.00018	0.00010	0.00524	0.0268
OTU106_*Bacteroidetes*_unclassified	0.00546	0.00000	0.00000	0.0345
OTU110_*Prevotellaceae*_unclassified	0.00002	0.00075	0.00436	0.0060
OTU111_*Methanomassiliicoccus*	0.00499	0.00003	0.00001	0.0017
OTU114_*Megasphaera*	0.00000	0.00287	0.00204	0.0046
OTU115_*Fibrobacter*	0.00000	0.00231	0.00257	0.0107
OTU119_*Prevotella*	0.00437	0.00000	0.00003	0.0006
OTU121_*Bacteroidales*_unclassified	0.00325	0.00031	0.00079	0.0321
OTU124_*Bacteroidetes*_unclassified	0.00426	0.00000	0.00003	0.0041
OTU126_*Anaeroplasma*	0.00420	0.00003	0.00001	0.0159
OTU127_*Acidaminococcus*	0.00002	0.00175	0.00244	0.0500
OTU128_*Bacteroidetes*_unclassified	0.00421	0.00000	0.00001	0.0041
OTU130_*Clostridiales*_unclassified	0.00420	0.00000	0.00000	0.0018
OTU131_*Bacteroidetes*_unclassified	0.00412	0.00000	0.00008	0.0057
OTU132_*Pyramidobacter*	0.00261	0.00059	0.00097	0.0043
OTU134_*Prevotella*	0.00404	0.00000	0.00000	0.0018
OTU137_*Escherichia.Shigella*	0.00195	0.00104	0.00089	0.0362
OTU138_*Prevotella*	0.00382	0.00000	0.00003	0.0009
OTU139_*Bacteroidetes*_unclassified	0.00385	0.00000	0.00000	0.0003
OTU140_*Anaerovibrio*	0.00003	0.00214	0.00157	0.0429
OTU141_*Prevotella*	0.00369	0.00000	0.00002	0.0057
OTU142_*Bacteroidales*_unclassified	0.00321	0.00049	0.00000	0.0373
OTU144_*Saccharofermentans*	0.00331	0.00022	0.00001	0.0408
OTU145_*Ruminococcaceae*_unclassified	0.00000	0.00000	0.00352	0.0085
OTU147_*Bacteroidales*_unclassified	0.00333	0.00000	0.00000	0.0003
OTU148_*Lachnospiraceae*_unclassified	0.00001	0.00251	0.00073	0.0045
OTU151_*Ruminococcaceae*_unclassified	0.00309	0.00001	0.00000	0.0007
OTU152_*Mitsuokella*	0.00002	0.00093	0.00211	0.0117
OTU153_*Firmicutes*_unclassified	0.00302	0.00000	0.00000	0.0085
OTU154_*Methanomassiliicoccus*	0.00301	0.00001	0.00001	0.0010
OTU158_*Clostridiales*_unclassified	0.00285	0.00006	0.00000	0.0009
OTU159_*Lachnospiraceae*_unclassified	0.00290	0.00000	0.00000	0.0085
OTU165_*Prevotellaceae*_unclassified	0.00000	0.00006	0.00264	0.0057
OTU167_*Veillonellaceae*_unclassified	0.00160	0.00093	0.00014	0.0347
OTU168_*Methanomicrobium*	0.00250	0.00013	0.00000	0.0009
OTU169_*Bacteroidetes*_unclassified	0.00261	0.00000	0.00000	0.0003
OTU173_*Megasphaera*	0.00000	0.00171	0.00083	0.0447
OTU174_*Bacteroidetes*_unclassified	0.00243	0.00007	0.00002	0.0227
OTU175_*Prevotella*	0.00195	0.00000	0.00057	0.0206
OTU176_*Prevotellaceae*_unclassified	0.00209	0.00035	0.00001	0.0050
OTU177_*Campylobacter*	0.00134	0.00054	0.00054	0.0391
OTU178_*Bacteroides*	0.00240	0.00000	0.00000	0.0003
OTU182_*Bacteroidetes*_unclassified	0.00211	0.00006	0.00015	0.0161
OTU183_*Bacteroidetes*_unclassified	0.00222	0.00008	0.00001	0.0096
OTU186_*Prevotella*	0.00001	0.00163	0.00064	0.0075

Table S6. *Cont.*

OTU190_*Sphaerochaeta*	0.00198	0.00022	0.00005	0.0038
OTU191_*Lachnospiraceae*_unclassified	0.00001	0.00077	0.00146	0.0162
OTU192_*Methanimicrococcus*	0.00220	0.00000	0.00000	0.0003
OTU193_*Bacteroidetes*_unclassified	0.00215	0.00000	0.00000	0.0018
OTU205_*Firmicutes*_unclassified	0.00000	0.00007	0.00176	0.0090
OTU206_*Lachnospiraceae*_unclassified	0.00131	0.00012	0.00041	0.0110
OTU208_*Bacteria*_unclassified	0.00180	0.00000	0.00000	0.0003
OTU209_*Parabacteroides*	0.00175	0.00000	0.00000	0.0085
OTU210_*Desulfovibrio*	0.00155	0.00012	0.00004	0.0112
OTU213_*Clostridiales*_unclassified	0.00166	0.00000	0.00000	0.0345
OTU216_*Ruminococcaceae*_unclassified	0.00136	0.00025	0.00000	0.0025
OTU217_*Bacteroidetes*_unclassified	0.00160	0.00000	0.00000	0.0003
OTU218_*Treponema*	0.00159	0.00000	0.00000	0.0018
OTU219_*Bacteroidetes*_unclassified	0.00155	0.00003	0.00000	0.0009
OTU220_*Prevotella*	0.00000	0.00058	0.00099	0.0227
OTU221_*Pseudobutyrivibrio*	0.00012	0.00001	0.00142	0.0108
OTU222_*Firmicutes*_unclassified	0.00153	0.00000	0.00002	0.0061
OTU223_*Ruminococcaceae*_unclassified	0.00155	0.00000	0.00000	0.0018
OTU224_*Prevotella*	0.00000	0.00090	0.00064	0.0473
OTU226_*Prevotella*	0.00138	0.00000	0.00015	0.0488
OTU228_*Subdivision3_bacteria_incertae_sedis*	0.00151	0.00000	0.00000	0.0018
OTU230_*Bacteroidetes*_unclassified	0.00001	0.00030	0.00118	0.0448
OTU232_*Lachnospiraceae*_unclassified	0.00147	0.00000	0.00000	0.0085
OTU239_*Bacteroidales*_unclassified	0.00001	0.00044	0.00091	0.0295
OTU245_*Clostridiales*_unclassified	0.00122	0.00000	0.00000	0.0003
OTU246_*Bacteroidetes*_unclassified	0.00102	0.00019	0.00001	0.0339
OTU248_*Desulfovibrionaceae*_unclassified	0.00104	0.00016	0.00000	0.0078
OTU249_*Bacteroides*	0.00119	0.00000	0.00000	0.0085
OTU252_*Treponema*	0.00000	0.00117	0.00001	0.0396
OTU255_*Comamonas*	0.00114	0.00000	0.00001	0.0061
OTU258_*Olsenella*	0.00000	0.00073	0.00036	0.0101
OTU262_*Firmicutes*_unclassified	0.00021	0.00082	0.00000	0.0180
OTU263_*Lachnospiraceae*_unclassified	0.00104	0.00000	0.00000	0.0345
OTU265_*Clostridiales*_unclassified	0.00102	0.00000	0.00000	0.0085
OTU266_*Porphyromonadaceae*_unclassified	0.00102	0.00000	0.00000	0.0003
OTU268_*Mitsuokella*	0.00001	0.00033	0.00066	0.0377
OTU269_*Ruminococcus*	0.00000	0.00044	0.00057	0.0106
OTU270_*Bibersteinia*	0.00088	0.00004	0.00003	0.0326
OTU273_*Prevotella*	0.00052	0.00000	0.00041	0.0463
OTU275_*Saccharofermentans*	0.00093	0.00000	0.00000	0.0018
OTU277_*Lachnospiracea_incertae_sedis*	0.00000	0.00000	0.00092	0.0345
OTU278_*Paraprevotella*	0.00075	0.00006	0.00009	0.0276
OTU279_*Clostridiales*_unclassified	0.00090	0.00000	0.00000	0.0003
OTU281_*Bacteroidetes*_unclassified	0.00089	0.00000	0.00000	0.0003
OTU282_*Lachnospiraceae*_unclassified	0.00000	0.00024	0.00064	0.0250
OTU287_*Pasteurellaceae*_unclassified	0.00078	0.00004	0.00003	0.0071
OTU290_*Bacteroidetes*_unclassified	0.00082	0.00000	0.00000	0.0003
OTU296_*Clostridiales*_unclassified	0.00075	0.00000	0.00001	0.0006
OTU298_*Pyramidobacter*	0.00071	0.00004	0.00000	0.0016

Table S6. *Cont.*

OTU306_*Megasphaera*	0.00000	0.00023	0.00048	0.0246
OTU310_*Treponema*	0.00068	0.00000	0.00000	0.0018
OTU311_*Clostridiales*_unclassified	0.00002	0.00050	0.00015	0.0182
OTU313_*Methanomassiliicoccus*	0.00062	0.00003	0.00000	0.0009
OTU314_*Blautia*	0.00000	0.00019	0.00046	0.0179
OTU320_*Alloprevotella*	0.00061	0.00000	0.00000	0.0018
OTU322_*Allisonella*	0.00000	0.00030	0.00029	0.0248
OTU325_*Bacteroides*	0.00037	0.00010	0.00011	0.0177
OTU326_*Ruminococcaceae*_unclassified	0.00054	0.00003	0.00000	0.0078
OTU327_*Clostridium*.XlVa	0.00046	0.00003	0.00007	0.0055
OTU328_*Ethanoligenens*	0.00056	0.00000	0.00000	0.0085
OTU329_*Methanomassiliicoccus*	0.00056	0.00000	0.00000	0.0018
OTU330_*Firmicutes*_unclassified	0.00056	0.00000	0.00000	0.0003
OTU333_*Ruminococcaceae*_unclassified	0.00036	0.00019	0.00000	0.0138
OTU337_*Bacteroidetes*_unclassified	0.00050	0.00000	0.00001	0.0249
OTU338_*Prevotella*	0.00040	0.00012	0.00000	0.0476
OTU340_*Intestinimonas*	0.00024	0.00025	0.00003	0.0221
OTU342_*Lachnospiracea_incertae_sedis*	0.00037	0.00013	0.00000	0.0137
OTU343_*Prevotellaceae*_unclassified	0.00050	0.00000	0.00000	0.0018
OTU344_*Firmicutes*_unclassified	0.00048	0.00000	0.00000	0.0345
OTU346_*Bacteroidetes*_unclassified	0.00048	0.00000	0.00000	0.0085
OTU348_*Mannheimia*	0.00045	0.00001	0.00000	0.0079
OTU349_*Bacteroidetes*_unclassified	0.00038	0.00008	0.00000	0.0021
OTU352_*Bacteroidetes*_unclassified	0.00040	0.00005	0.00000	0.0090
OTU353_*Bacteroidetes*_unclassified	0.00044	0.00000	0.00000	0.0345
OTU360_*Bacteroidetes*_unclassified	0.00000	0.00007	0.00036	0.0377
OTU363_*Firmicutes*_unclassified	0.00035	0.00000	0.00006	0.0034
OTU364_*Ruminococcaceae*_unclassified	0.00017	0.00000	0.00023	0.0181
OTU365_*Neisseriaceae*_unclassified	0.00038	0.00001	0.00001	0.0010
OTU366_*Bacteroidetes*_unclassified	0.00039	0.00000	0.00000	0.0082
OTU370_*Clostridiales*_unclassified	0.00039	0.00000	0.00000	0.0345
OTU371_*Sphaerochaeta*	0.00035	0.00003	0.00001	0.0063
OTU372_*Bacteria*_unclassified	0.00037	0.00001	0.00000	0.0217
OTU373_*Clostridiales*_unclassified	0.00023	0.00015	0.00000	0.0181
OTU382_*Prevotellaceae*_unclassified	0.00000	0.00000	0.00035	0.0345
OTU383_*Bacteria*_unclassified	0.00035	0.00000	0.00000	0.0345
OTU385_*Moraxella*	0.00033	0.00001	0.00000	0.0040
OTU387_*Bacteroidetes*_unclassified	0.00035	0.00000	0.00000	0.0003
OTU389_*Bacteroidetes*_unclassified	0.00034	0.00000	0.00000	0.0345
OTU391_*Lachnospiraceae*_unclassified	0.00000	0.00022	0.00012	0.0233
OTU392_*Bilophila*	0.00033	0.00000	0.00000	0.0003
OTU393_*Sphaerochaeta*	0.00027	0.00006	0.00000	0.0089
OTU396_*Proteobacteria*_unclassified	0.00000	0.00001	0.00030	0.0061
OTU402_*Firmicutes*_unclassified	0.00030	0.00000	0.00000	0.0085
OTU403_*Treponema*	0.00028	0.00002	0.00000	0.0023
OTU406_*Proteobacteria*_unclassified	0.00030	0.00000	0.00000	0.0085
OTU411_*Clostridia*_unclassified	0.00028	0.00000	0.00000	0.0003
OTU412_*Treponema*	0.00028	0.00000	0.00000	0.0345
OTU413_*Clostridium*.XlVb	0.00023	0.00003	0.00001	0.0031

Table S6. *Cont.*

OTU415_*Dorea*	0.00000	0.00024	0.00003	0.0078
OTU416_*Bacteroidetes*_unclassified	0.00027	0.00000	0.00000	0.0085
OTU418_*Bacteroidetes*_unclassified	0.00027	0.00000	0.00000	0.0018
OTU421_*Sphaerochaeta*	0.00019	0.00008	0.00000	0.0045
OTU422_*Porphyromonadaceae*_unclassified	0.00027	0.00000	0.00000	0.0003
OTU430_*Clostridiales*_unclassified	0.00026	0.00000	0.00000	0.0018
OTU443_*Subdivision5_bacteria_incertae_sedis*	0.00023	0.00000	0.00000	0.0018
OTU447_*Firmicutes*_unclassified	0.00019	0.00000	0.00004	0.0037
OTU449_*Alysiella*	0.00022	0.00000	0.00000	0.0018
OTU454_*Bacteroidetes*_unclassified	0.00021	0.00000	0.00000	0.0018
OTU459_*Ruminococcaceae*_unclassified	0.00021	0.00000	0.00000	0.0018
OTU462_*Ruminococcaceae*_unclassified	0.00001	0.00011	0.00009	0.0236
OTU464_*Burkholderiales*_unclassified	0.00000	0.00006	0.00015	0.0030
OTU472_*Ruminococcaceae*_unclassified	0.00020	0.00000	0.00000	0.0018
OTU477_*Ruminococcaceae*_unclassified	0.00017	0.00000	0.00002	0.0008
OTU479_*Subdivision5_bacteria_incertae_sedis*	0.00019	0.00000	0.00000	0.0017
OTU480_*Alloprevotella*	0.00019	0.00000	0.00000	0.0085
OTU481_*Bacteroidetes*_unclassified	0.00019	0.00000	0.00000	0.0016
OTU484_*Kingella*	0.00018	0.00000	0.00000	0.0085
OTU487_*Firmicutes*_unclassified	0.00018	0.00000	0.00000	0.0003
OTU489_*Bacteria*_unclassified	0.00017	0.00000	0.00000	0.0345
OTU499_*Ruminococcaceae*_unclassified	0.00017	0.00000	0.00000	0.0345
OTU500_*Butyricimonas*	0.00015	0.00001	0.00001	0.0266
OTU501_*Bacteria*_unclassified	0.00016	0.00000	0.00000	0.0016
OTU502_*Moraxella*	0.00016	0.00000	0.00000	0.0018
OTU505_*Butyrivibrio*	0.00014	0.00000	0.00001	0.0039
OTU506_*Bacteria*_unclassified	0.00015	0.00000	0.00000	0.0003
OTU509_*Bacteroidales*_unclassified	0.00015	0.00000	0.00000	0.0084
OTU517_*Treponema*	0.00015	0.00000	0.00000	0.0085
OTU518_*Bacteroidales*_unclassified	0.00013	0.00001	0.00000	0.0039
OTU522_*Ruminococcaceae*_unclassified	0.00014	0.00000	0.00000	0.0345
OTU529_*Bacteria*_unclassified	0.00012	0.00001	0.00000	0.0118
OTU531_*Ruminococcaceae*_unclassified	0.00011	0.00001	0.00001	0.0489
OTU532_*Bacteroidetes*_unclassified	0.00000	0.00000	0.00013	0.0342
OTU533_*Ruminococcaceae*_unclassified	0.00013	0.00000	0.00000	0.0345
OTU537_*Sphaerochaeta*	0.00013	0.00000	0.00000	0.0018
OTU539_*Clostridiales*_unclassified	0.00000	0.00004	0.00008	0.0044
OTU540_*Bacteroidetes*_unclassified	0.00012	0.00000	0.00001	0.0217
OTU541_*Parabacteroides*	0.00012	0.00000	0.00000	0.0345
OTU545_*Clostridiales*_unclassified	0.00012	0.00000	0.00000	0.0085
OTU546_*Oligosphaera*	0.00010	0.00002	0.00001	0.0343
OTU549_*Alistipes*	0.00012	0.00000	0.00000	0.0017
OTU553_*Bacteria*_unclassified	0.00012	0.00000	0.00000	0.0085
OTU554_*Bacteroidales*_unclassified	0.00010	0.00001	0.00000	0.0371
OTU556_*Clostridiales*_unclassified	0.00011	0.00001	0.00000	0.0249
OTU559_*Ruminococcaceae*_unclassified	0.00011	0.00000	0.00000	0.0085
OTU563_*Oxalobacteraceae*_unclassified	0.00011	0.00000	0.00000	0.0082
OTU566_*Porphyromonadaceae*_unclassified	0.00010	0.00000	0.00001	0.0046
OTU569_*Clostridiales*_unclassified	0.00010	0.00000	0.00001	0.0010

Table S6. *Cont.*

OTU576_*Chryseobacterium*	0.00008	0.00001	0.00001	0.0241
OTU577_*Bacteroidetes*_unclassified	0.00010	0.00000	0.00000	0.0345
OTU579_*Ruminococcaceae*_unclassified	0.00009	0.00000	0.00000	0.0345
OTU597_*Clostridiales*_unclassified	0.00008	0.00000	0.00000	0.0345
OTU598_*Streptococcus*	0.00008	0.00000	0.00000	0.0345
OTU601_*Ruminococcaceae*_unclassified	0.00008	0.00000	0.00000	0.0082
OTU603_*Lachnospiraceae*_unclassified	0.00006	0.00001	0.00000	0.0101
OTU617_*Bacteroidetes*_unclassified	0.00008	0.00000	0.00000	0.0017
OTU619_*Pasteurellaceae*_unclassified	0.00007	0.00000	0.00001	0.0279
OTU624_*Ruminococcaceae*_unclassified	0.00000	0.00000	0.00007	0.0342
OTU625_*Bacteria*_unclassified	0.00007	0.00000	0.00000	0.0342
OTU626_*Spirochaetales*_unclassified	0.00000	0.00000	0.00007	0.0345
OTU629_*Neisseriaceae*_unclassified	0.00007	0.00000	0.00000	0.0085
OTU630_*Subdivision5_bacteria_incertae_sedis*	0.00007	0.00000	0.00000	0.0345
OTU637_*Porphyromonas*	0.00006	0.00000	0.00001	0.0055
OTU639_*Lachnospiraceae*_unclassified	0.00000	0.00000	0.00007	0.0345
OTU658_*Bacteria*_unclassified	0.00006	0.00000	0.00000	0.0082
OTU659_*Meganema*	0.00004	0.00001	0.00001	0.0491
OTU663_*Neisseriaceae*_unclassified	0.00006	0.00000	0.00000	0.0345
OTU678_*Lachnospiraceae*_unclassified	0.00000	0.00001	0.00005	0.0281
OTU688_*Moraxella*	0.00005	0.00000	0.00000	0.0345
OTU691_*Bacteria*_unclassified	0.00005	0.00000	0.00000	0.0345
OTU705_*Pasteurellaceae*_unclassified	0.00004	0.00000	0.00000	0.0334
OTU709_*Cardiobacteriaceae*_unclassified	0.00004	0.00000	0.00000	0.0084
OTU720_*Bacteroidetes*_unclassified	0.00004	0.00000	0.00000	0.0342
OTU729_*Fibrobacter*	0.00003	0.00000	0.00000	0.0342
OTU732_*Synergistes*	0.00003	0.00000	0.00000	0.0342
OTU767_*Victivallis*	0.00003	0.00000	0.00000	0.0342
OTU768_*Clostridiales*_unclassified	0.00000	0.00000	0.00003	0.0342
OTU770_*Streptococcus*	0.00003	0.00000	0.00000	0.0342
OTU771_*Peptostreptococcus*	0.00003	0.00000	0.00000	0.0342
OTU779_*Coriobacteriaceae*_unclassified	0.00003	0.00000	0.00000	0.0078
OTU782_*Brevundimonas*	0.00003	0.00000	0.00000	0.0078
OTU783_*Porphyromonas*	0.00003	0.00000	0.00000	0.0078
OTU807_*Corynebacterium*	0.00002	0.00000	0.00000	0.0334
OTU813_*Lachnospiraceae*_unclassified	0.00002	0.00000	0.00000	0.0334

Table S7 the top 50 features selected of RandomForest classification model based on MDA

Order based on MDA	MRO-MRC-MCA	MRO-MRC	MRO-MCA	MRC-MCA
1	OTU93_*Treponema*	OTU365_*Neisseriaceae*_unclassified	OTU216_*Ruminococcaceae*_unclassified	OTU74_*Treponema*
2	OTU87_*Butyrivibrio*	OTU296_*Clostridiales*_unclassified	OTU464_*Burkholderiales*_unclassified	OTU104_*Prevotella*
3	OTU539_*Clostridiales*_unclassified	OTU5_*Sphingobacteriaceae*_unclassified	OTU60_*Prevotella*	OTU396_*Proteobacteria*_unclassified
4	OTU330_*Firmicutes*_unclassified	OTU148_*Lachnospiraceae*_unclassified	OTU22_*Succiniclasticum*	OTU539_*Clostridiales*_unclassified
5	OTU313_*Methanomassiliicoccus*	OTU290_*Bacteroidetes*_unclassified	OTU422_*Porphyromonadaceae*_unclassified	OTU87_*Butyrivibrio*
6	OTU83_*Bacteroidales*_unclassified	OTU18_*Prevotella*	OTU39_*Ruminococcaceae*_unclassified	OTU83_*Bacteroidales*_unclassified
7	OTU89_*Bacteroidetes*_unclassified	OTU111_*Methanomassiliicoccus*	OTU94_*Clostridiales*_unclassified	OTU42_*Prevotella*
8	OTU396_*Proteobacteria*_unclassified	OTU411_*Clostridia*_unclassified	OTU178_*Bacteroides*	OTU93_*Treponema*
9	OTU75_*Ruminococcaceae*_unclassified	OTU569_*Clostridiales*_unclassified	OTU147_*Bacteroidales*_unclassified	OTU221_*Pseudobutyrivibrio*
10	OTU24_*Cloacibacillus*	OTU119_*Prevotella*	OTU290_*Bacteroidetes*_unclassified	OTU110_*Prevotellaceae*_unclassified
11	OTU154_*Methanomassiliicoccus*	OTU487_*Firmicutes*_unclassified	OTU349_*Bacteroidetes*_unclassified	OTU599_*Spartobacteria*_unclassified
12	OTU387_*Bacteroidetes*_unclassified	OTU447_*Firmicutes*_unclassified	OTU539_*Clostridiales*_unclassified	OTU100_*Prevotellaceae*_unclassified
13	OTU217_*Bacteroidetes*_unclassified	OTU281_*Bacteroidetes*_unclassified	OTU55_*Sphaerochaeta*	OTU163_*Prevotellaceae*_unclassified
14	OTU104_*Prevotella*	OTU422_*Porphyromonadaceae*_unclassified	OTU154_*Methanomassiliicoccus*	OTU54_*Prevotella*
15	OTU422_*Porphyromonadaceae*_unclassified	OTU154_*Methanomassiliicoccus*	OTU330_*Firmicutes*_unclassified	OTU432_*Lachnospiraceae*_unclassified
16	OTU74_*Treponema*	OTU217_*Bacteroidetes*_unclassified	OTU208_*Bacteria*_unclassified	OTU88_*Treponema*
17	OTU178_*Bacteroides*	OTU79_*Neisseriaceae*_unclassified	OTU313_*Methanomassiliicoccus*	OTU67_*Treponema*
18	OTU5_*Sphingobacteriaceae*_unclassified	OTU24_*Cloacibacillus*	OTU89_*Bacteroidetes*_unclassified	OTU643_*Neisseria*
19	OTU290_*Bacteroidetes*_unclassified	OTU208_*Bacteria*_unclassified	OTU411_*Clostridia*_unclassified	OTU177_*Campylobacter*
20	OTU55_*Sphaerochaeta*	OTU114_*Megasphaera*	OTU24_*Cloacibacillus*	OTU145_*Ruminococcaceae*_unclassified
21	OTU39_*Ruminococcaceae*_unclassified	OTU506_*Bacteria*_unclassified	OTU413_*Clostridium.XlVb*	OTU205_*Firmicutes*_unclassified
22	OTU90_*Bacteroidetes*_unclassified	OTU330_*Firmicutes*_unclassified	OTU296_*Clostridiales*_unclassified	OTU273_*Prevotella*
23	OTU94_*Clostridiales*_unclassified	OTU75_*Ruminococcaceae*_unclassified	OTU403_*Treponema*	OTU15_*Prevotellaceae*_unclassified

Table S7. *Cont.*

24	OTU365_*Neisseriaceae*_unclassified	OTU94_*Clostridiales*_unclassified	OTU266_*Porphyromonadaceae*_unclassified	OTU314_*Blautia*
25	OTU411_*Clostridia*_unclassified	OTU313_*Methanomassiliicoccus*	OTU298_*Pyramidobacter*	OTU48_*Treponema*
26	OTU296_*Clostridiales*_unclassified	OTU387_*Bacteroidetes*_unclassified	OTU5_*Sphingobacteriaceae*_unclassified	OTU591_*Atopobium*
27	OTU487_*Firmicutes*_unclassified	OTU147_*Bacteroidales*_unclassified	OTU281_*Bacteroidetes*_unclassified	OTU415_*Dorea*
28	OTU147_*Bacteroidales*_unclassified	OTU178_*Bacteroides*	OTU1_*Sphingobacteriaceae*_unclassified	OTU123_*Prevotella*
29	OTU99_*Elusimicrobium*	OTU139_*Bacteroidetes*_unclassified	OTU151_*Ruminococcaceae*_unclassified	OTU52_*Bacteria*_unclassified
30	OTU392_*Bilophila*	OTU477_*Ruminococcaceae*_unclassified	OTU111_*Methanomassiliicoccus*	OTU171_*Olsenella*
31	OTU506_*Bacteria*_unclassified	OTU93_*Treponema*	OTU119_*Prevotella*	OTU143_*Fibrobacter*
32	OTU169_*Bacteroidetes*_unclassified	OTU363_*Firmicutes*_unclassified	OTU217_*Bacteroidetes*_unclassified	OTU6_*Prevotella*
33	OTU119_*Prevotella*	OTU39_*Ruminococcaceae*_unclassified	OTU34_*Ruminococcus*	OTU611_*Bacteria*_unclassified
34	OTU279_*Clostridiales*_unclassified	OTU169_*Bacteroidetes*_unclassified	OTU365_*Neisseriaceae*_unclassified	OTU115_*Fibrobacter*
35	OTU139_*Bacteroidetes*_unclassified	OTU62_*Prevotellaceae*_unclassified	OTU387_*Bacteroidetes*_unclassified	OTU165_*Prevotellaceae*_unclassified
36	OTU221_*Pseudobutyrivibrio*	OTU279_*Clostridiales*_unclassified	OTU83_*Bacteroidales*_unclassified	OTU29_*Prevotella*
37	OTU208_*Bacteria*_unclassified	OTU266_*Porphyromonadaceae*_unclassified	OTU392_*Bilophila*	OTU96_*Alloprevotella*
38	OTU266_*Porphyromonadaceae*_unclassified	OTU33_*Bacteroidetes*_unclassified	OTU6_*Prevotella*	OTU86_*Pyramidobacter*
39	OTU33_*Bacteroidetes*_unclassified	OTU392_*Bilophila*	OTU219_*Bacteroidetes*_unclassified	OTU91_*Prevotellaceae*_unclassified
40	OTU245_*Clostridiales*_unclassified	OTU89_*Bacteroidetes*_unclassified	OTU33_*Bacteroidetes*_unclassified	OTU140_*Anaerovibrio*
41	OTU110_*Prevotellaceae*_unclassified	OTU86_*Pyramidobacter*	OTU75_*Ruminococcaceae*_unclassified	OTU768_*Clostridiales*_unclassified
42	OTU111_*Methanomassiliicoccus*	OTU99_*Elusimicrobium*	OTU192_*Methanimicrococcus*	OTU698_*Parasutterella*
43	OTU281_*Bacteroidetes*_unclassified	OTU245_*Clostridiales*_unclassified	OTU190_*Sphaerochaeta*	OTU313_*Methanomassiliicoccus*
44	OTU192_*Methanimicrococcus*	OTU55_*Sphaerochaeta*	OTU421_*Sphaerochaeta*	OTU626_*Spirochaetales*_unclassified
45	OTU42_*Prevotella*	OTU90_*Bacteroidetes*_unclassified	OTU13_*Prevotella*	OTU78_*Bacteroidales*_unclassified
46	OTU79_*Neisseriaceae*_unclassified	OTU192_*Methanimicrococcus*	OTU115_*Fibrobacter*	OTU524_*Lachnospiraceae*_unclassified
47	OTU477_*Ruminococcaceae*_unclassified	OTU138_*Prevotella*	OTU168_*Methanomicrobium*	OTU464_*Burkholderiales*_unclassified
48	OTU6_*Prevotella*	OTU168_*Methanomicrobium*	OTU487_*Firmicutes*_unclassified	OTU66_*Treponema*

Table S7. *Cont.*

49	OTU60_*Prevotella*	OTU60_*Prevotella*	OTU506_*Bacteria*_unclassified	OTU596_*Ruminococcaceae*_unclassified
50	OTU158_*Clostridiales*_unclassified	OTU186_*Prevotella*	OTU40_*Bacteroidetes*_unclassified	OTU382_*Prevotellaceae*_unclassified

Table S8. The prediction accuracy of Random Forest regression model

Items	Prediction accuracy (%)
CP	73.01
NDF	73.79
NFC	75.81
acetate	61.40
propionate	40.95
butyrate	57.03
valerate	73.05
Total VFA	70.06
Slaughter weight	69.50

The Random Forest regression model were used to select the rumen bacteria that were important for intake of major nutrient (CP, NDF and NFC). The model was run in R software using randomForest pachkage (v 4.6-14) with 10000 ntrees. The percent variance explained was reported for the estimation of accuracy of regression model. CP: Crude protein average daily intake; NDF: Neutral detergent fibers average daily intake; NFC: Non-fibrous carbohydrates average daily intake, Total VFA: Total volatile fatty acids.

Supplementary Figures

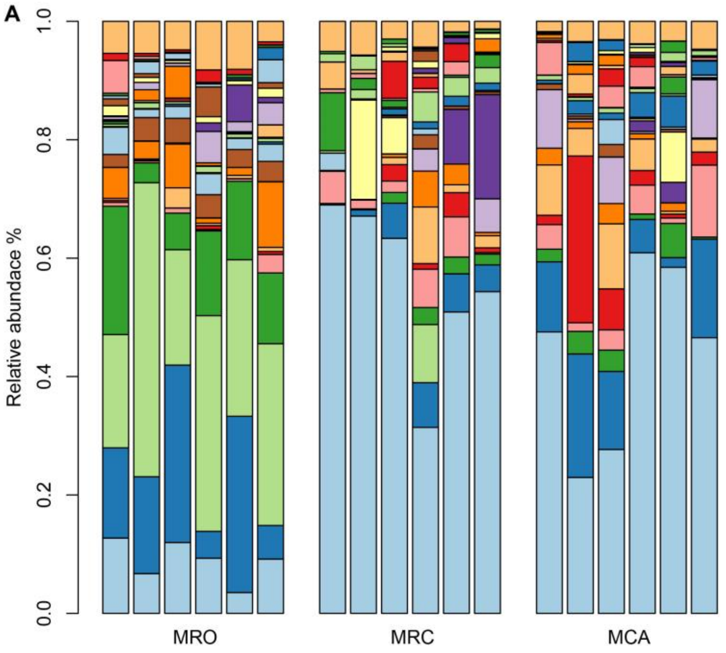

Figure S1. *Cont.*

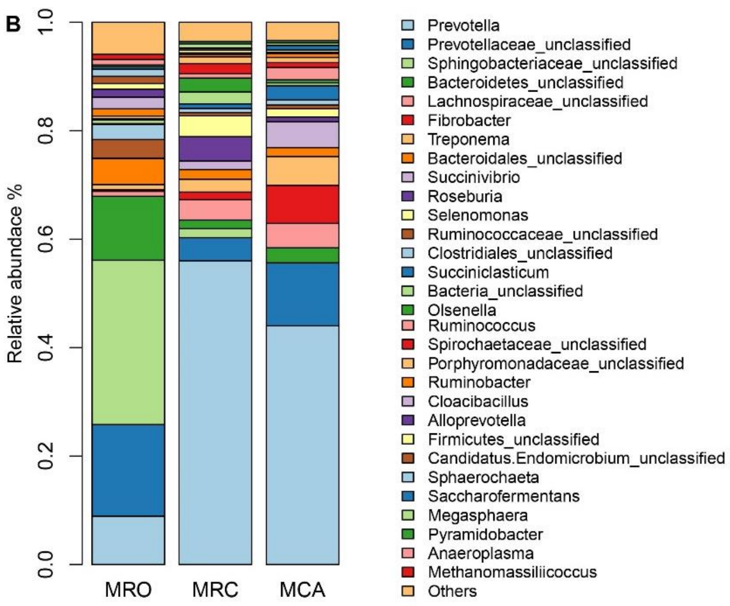

Figure S1. The rumen microbiome across three groups at genus level. Each bar shows the relative abundance of individual (**A**) or average (**B**) samples collected at MRO, MRC and MCA.

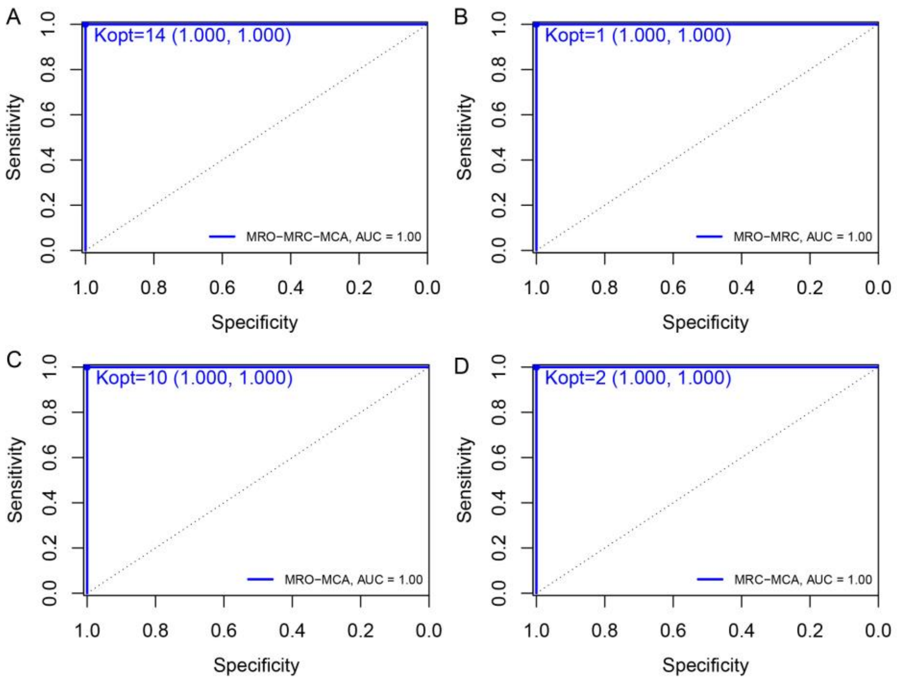

Figure S2. AUC curve of RandomForest classification model for differentiating MRO, MRC and MCA based on mean decrease accuracy: A: three groups; B: MRO vs MRC; C: MRO vs MCA; D: MRC vs MCA. The ‘Kopt’ shows the number of optimal variables fitted the AUCRF model. The values in parentheses are (specificity, sensitivity).

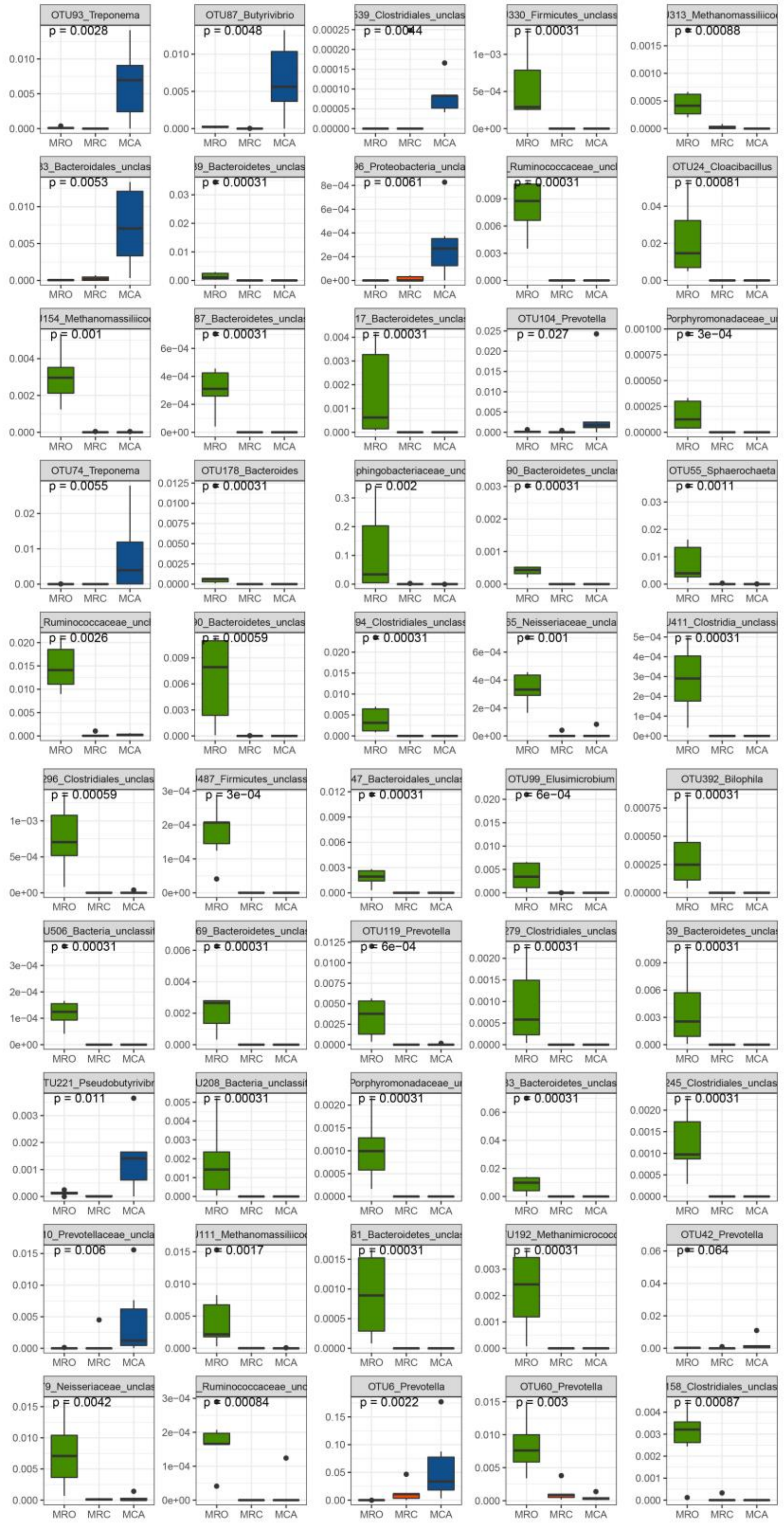

Figure S3. Top 50 features boxplots identified by AUCRF that differentiate MRO, MRC and MCA.The *p* values were calculated by using Kruskal–Wallis test. MRO = milk replacer, MRC = milk replacer + concentrate, MCA = milk replacer + concentrate + alfalfa.

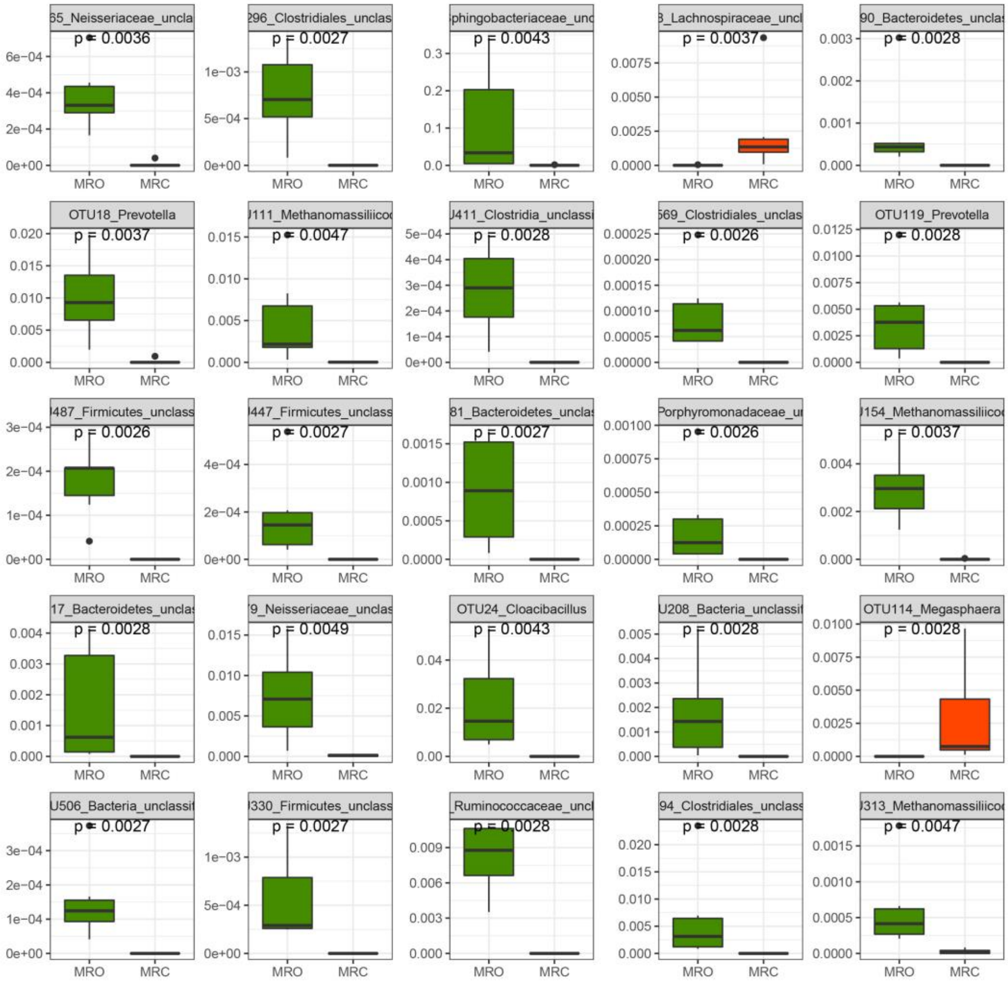

Figure S4. Top 25 predictors of pairwise AUCRF for differentiating MRO-MRC. The *p* values were calculated by using Kruskal–Wallis test. MRO = milk replacer, MRC = milk replacer + concentrate.

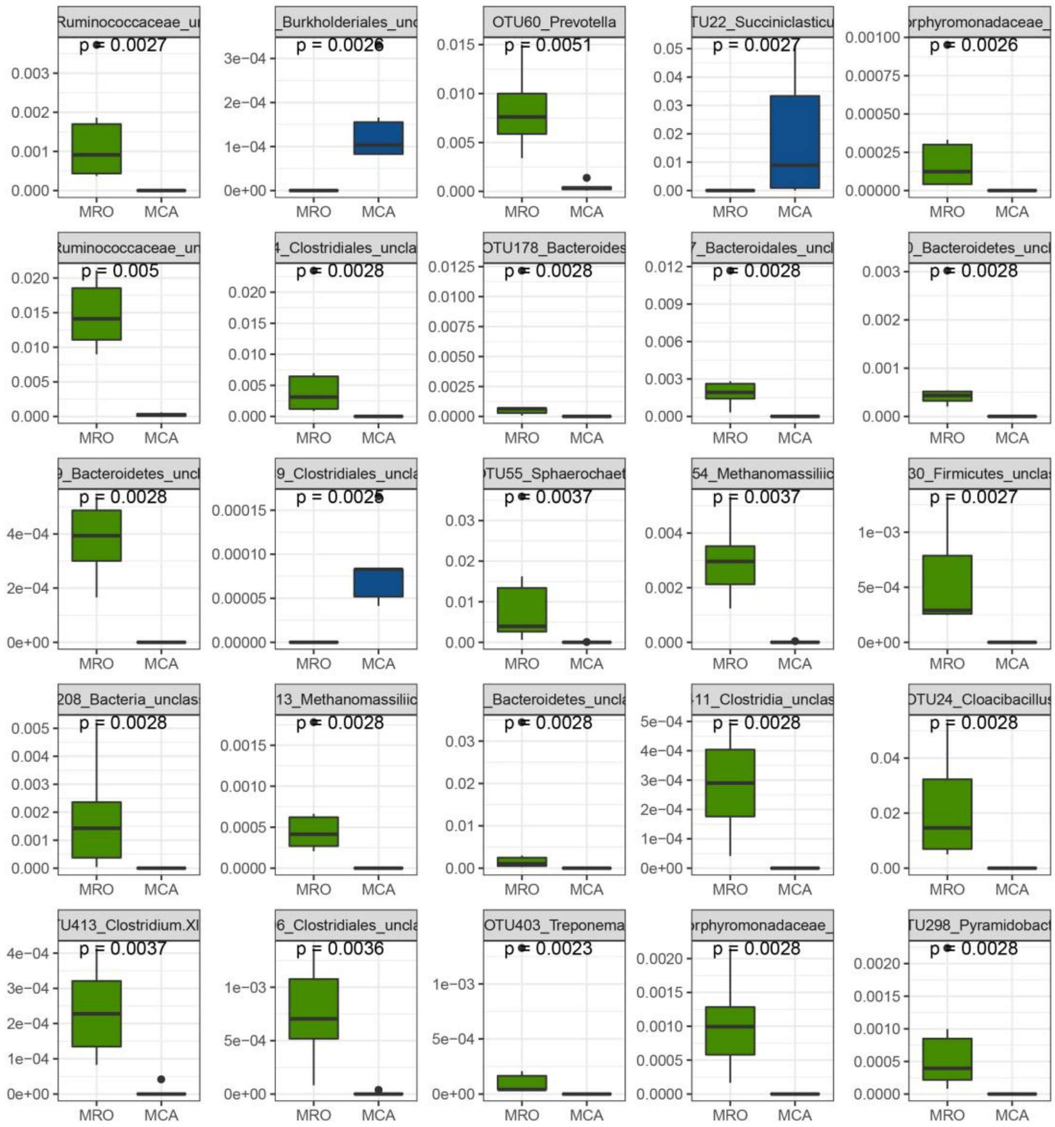

Figure S5. Top 25 predictors of pairwise AUCRF for differentiating MRO and MCA. The *p* values were calculated by using Kruskal–Wallis test. MRO = milk replacer, MCA = milk replacer + concentrate + alfalfa.

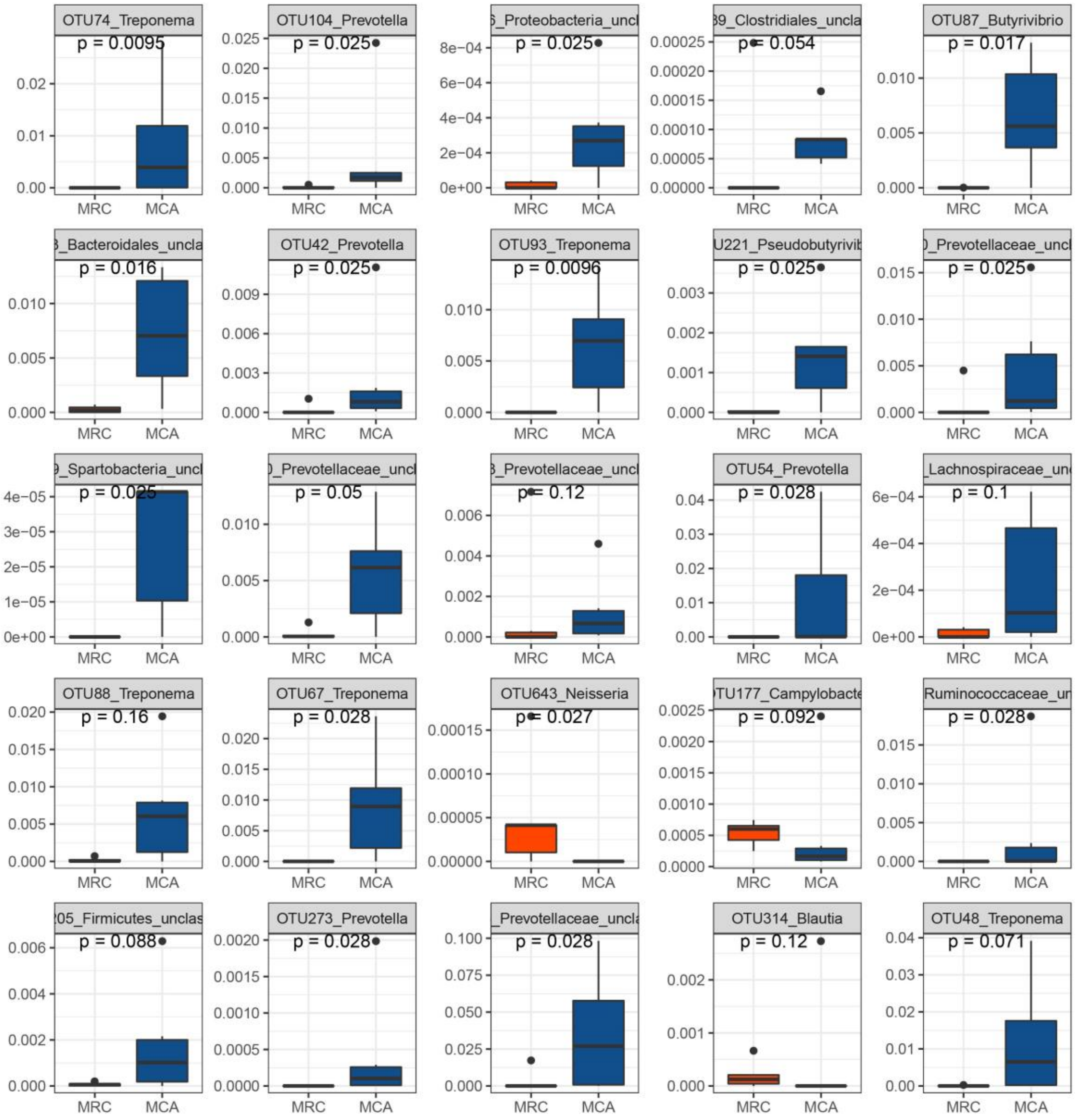

Figure S6. Top 25 predictors of pairwise AUCRF for differentiating MRC-MCA. The *p* values were calculated by using Kruskal–Wallis test. MRC = milk replacer + concentrate, MCA = milk replacer + concentrate + alfalfa.

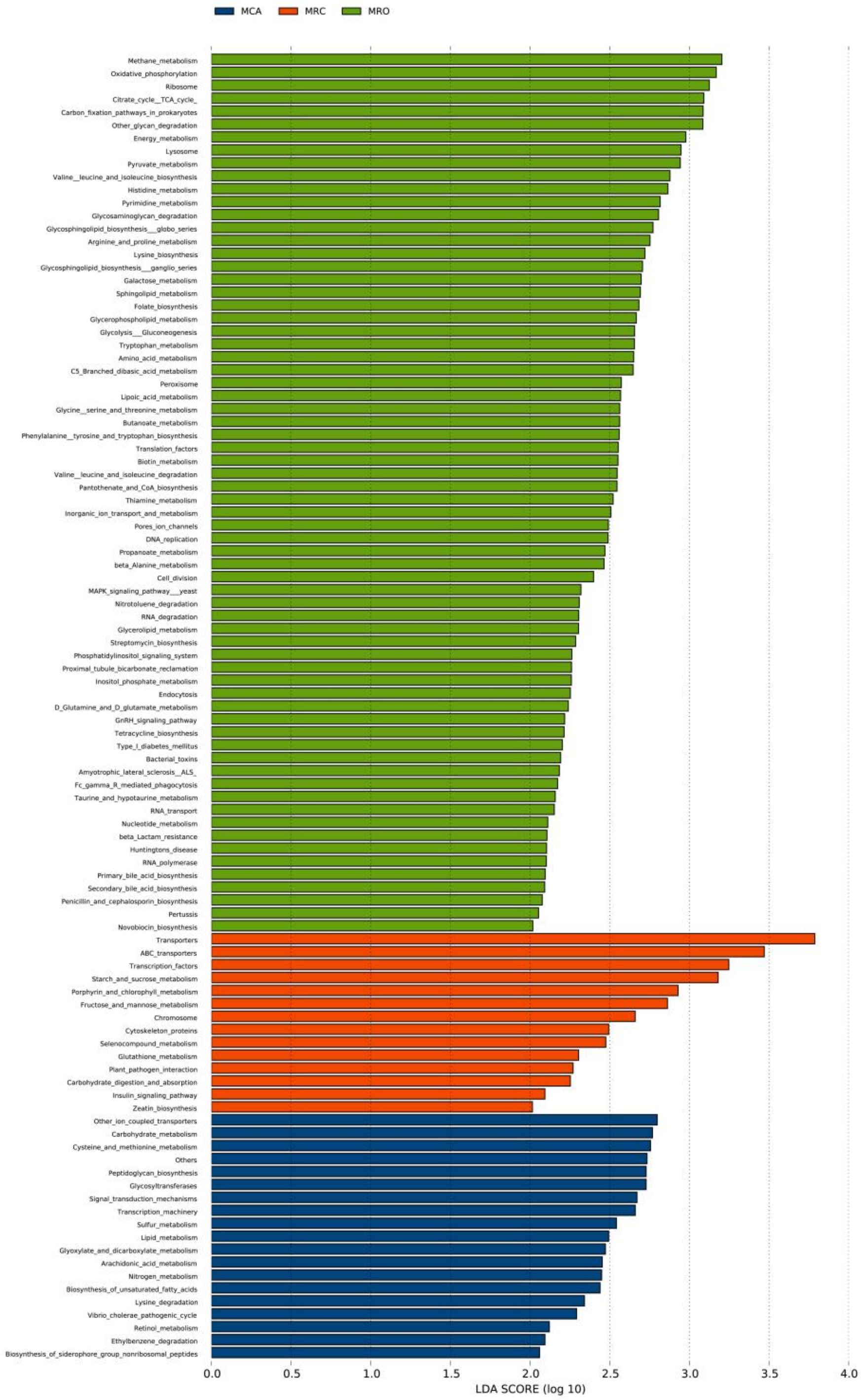

Figure S7. Predictive function analysis (levels 3 PICRUSt)

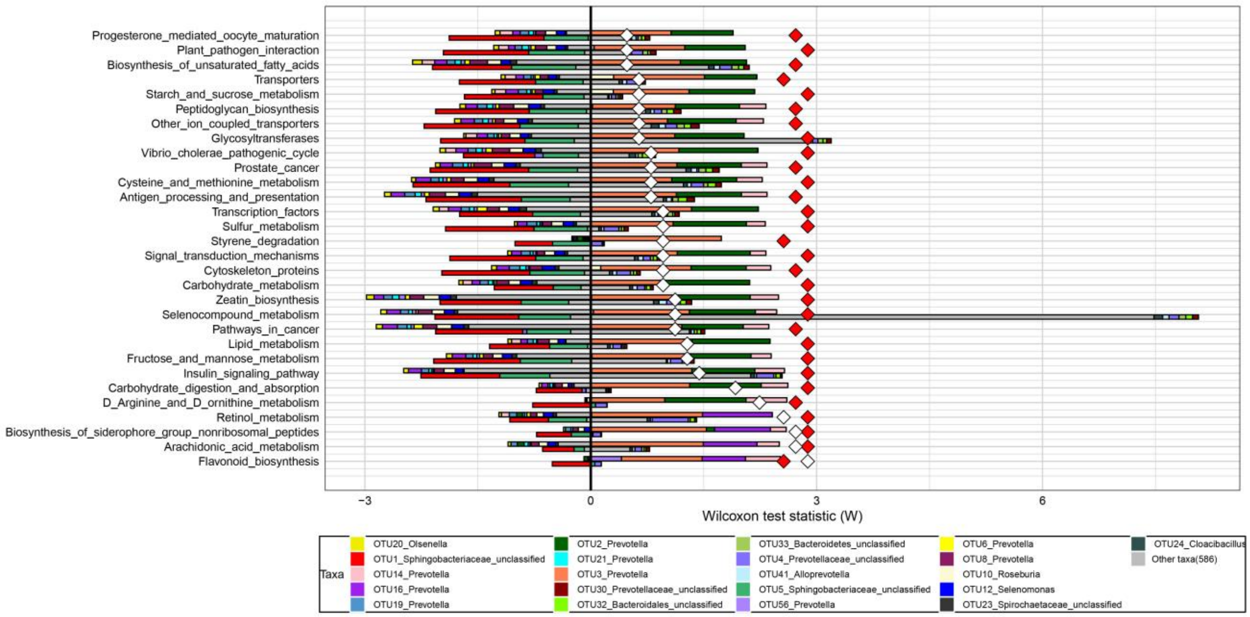

Figure S8. Comparing taxon-level contribution profiles of functional shifts in MRC rumen samples by FishTaco. Taxon-level shift contribution profiles for some case-associated (MRC case, MRO control) functional modules by FishTaco. The horizontal axis represents rank and statistic scores, and the vertical axis represents related pathways. For each functional pathway, the bar on the top-right of Y axis represents case-associated bacteria driving the enrichment in the functional module; the bar on the top-left of Y axis indicates case-associated bacteria attenuating functional shift; the bar on the bottom-right of Y axis represents bacteria depleted in control driving functional shift; the bar on the bottom-left of Y axis shows bacteria depleted in control attenuating functional shift. White diamonds represent bacterial-based functional shift scores. FishTaco:Functional Shifts' Taxonomic Contributors; MRO = milk replacer; MRC = milk replacer + concentrate

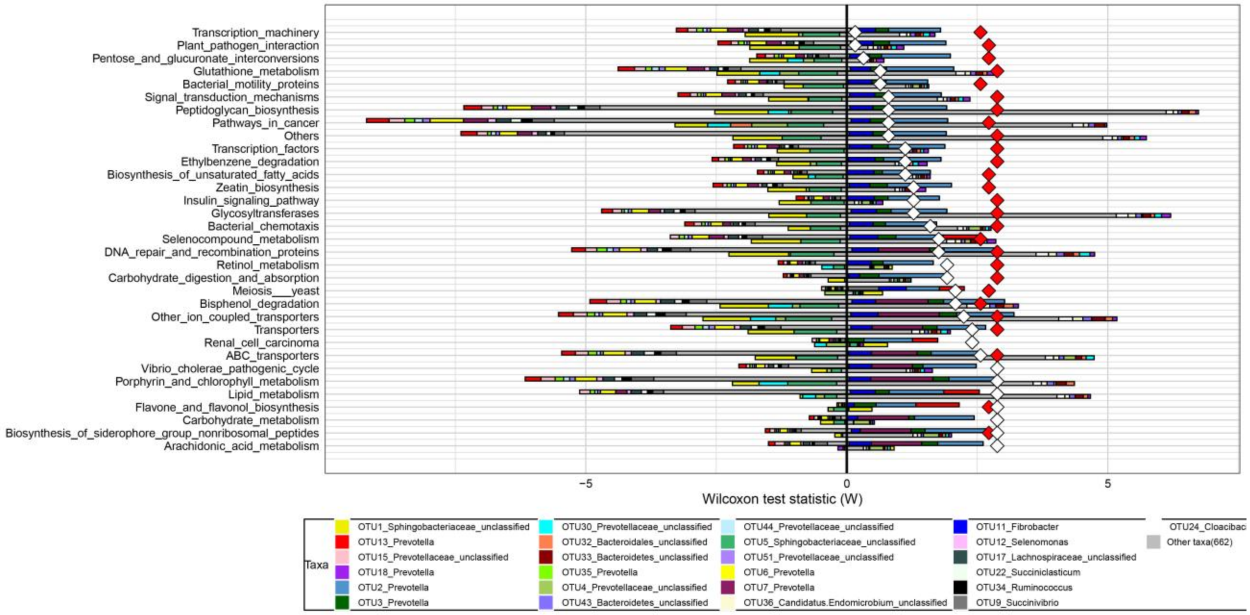

Figure S9. Comparing taxon-level contribution profiles of functional shifts in MCA rumen samples by FishTaco. Taxon-level shift contribution profiles for some case-associated (MCA case, MRO control) functional modules by Fish Taco. The horizontal axis represents rank and statistic scores, and the vertical axis represents related pathways. For each functional pathway, the bar on the top-right of Y axis represents case-associated bacteria driving the enrichment in the functional module; the bar on the top-left of Y axis indicates case-associated bacteria attenuating functional shift; the bar on the bottom-right of Y axis represents bacteria depleted in control driving functional shift; the bar on the bottom-left of Y axis shows bacteria depleted in control attenuating functional shift. White diamonds represent bacterial-based functional shift scores. MRO = milk replacer; MCA = milk replacer + concentrate + alfalfa; Fish Taco:Functional Shifts' Taxonomic Contributors;

Supplemental File legends

File S1: Pearson correlation between top 50 species selected by RandomForest regression and major nutrient intake of goat kids (sheet 'ADFI') and fermentation parameters and pre-slaughter live weight of goat kids (sheet 'VFA'). We used heatmap color to label the pearson coefficients and pvalue. Also, the NCBI Blast results were shown in the table.

.

File S2: OTUs taxonomy, their NCBI Blast results and sequences

Supplementary References

1. AOAC. Official methods of analysis. 15th ed. Washington, DC:Association of Official Analytical Chemists; 1990.
2. Xiaokang, L.; Xie, B.; Huang W.; Wang S.; Bi, Y.; Tao, H.; Cui K; Diao, Q.; Zhang N. Effects of early feeding on rumen and small intestine morphology of goat kids. *Acta Vet. Et Zootech. Sin.* **2019**, *50*, 1006–1015, doi: 10.11843/j.issn.0366-6964.2019.05.011.
3. Calle, M.L.; Urrea, V.; Boulesteix, A.L.; Malats, N. AUC-RF: a new strategy for genomic profiling with random forest. *Hum Hered* **2011**, *72*, 121–132, doi: 10.1159/000330778.
4. Langille, M.G.I.; Zaneveld, J.; Caporaso, J.G.; McDonald, D.; Knights, D.; Reyes, J.A.; Clemente, J.C.; Burkepile, D.E.; Thurber, R.L.V.; Knight, R.; et al. Predictive functional profiling of microbial communities using 16S rRNA marker gene sequences. *Nat Biotechnol* **2013**, *31*, 814, doi: 10.1038/nbt.2676.
